# Multiscale deep learning framework captures systemic immune features in lymph nodes predictive of triple negative breast cancer outcome in large‐scale studies

**DOI:** 10.1002/path.6088

**Published:** 2023-05-25

**Authors:** Gregory Verghese, Mengyuan Li, Fangfang Liu, Amit Lohan, Nikhil Cherian Kurian, Swati Meena, Patrycja Gazinska, Aekta Shah, Aasiyah Oozeer, Terry Chan, Mark Opdam, Sabine Linn, Cheryl Gillett, Elena Alberts, Thomas Hardiman, Samantha Jones, Selvam Thavaraj, J Louise Jones, Roberto Salgado, Sarah E Pinder, Swapnil Rane, Amit Sethi, Anita Grigoriadis

**Affiliations:** ^1^ Cancer Bioinformatics, School of Cancer & Pharmaceutical Sciences, Faculty of Life Sciences and Medicine King's College London London UK; ^2^ School of Cancer & Pharmaceutical Sciences, Faculty of Life Sciences and Medicine King's College London London UK; ^3^ Breast Cancer Now Unit, School of Cancer and Pharmaceutical Sciences, Faculty of Life Sciences and Medicine King's College London London UK; ^4^ Tianjin Medical University Cancer Institute and Hospital, National Clinical Research Center for Cancer, Tianjin's Clinical Research Center for Cancer, Key Laboratory of Breast Cancer Prevention and Therapy, Tianjin Medical University, Ministry of Education Key Laboratory of Cancer Prevention and Therapy Tianjin PR China; ^5^ Department of Electrical Engineering Indian Institute of Technology Bombay Mumbai India; ^6^ Biobank Research Group Lukasiewicz Research Network, PORT Polish Center for Technology Development Wroclaw Poland; ^7^ Department of Pathology Tata Memorial Centre, Tata Memorial Hospital, Homi Bhabha National Institute Mumbai India; ^8^ King's Health Partners Cancer Biobank, King's College London London UK; ^9^ Division of Molecular Pathology The Netherlands Cancer Institute Amsterdam The Netherlands; ^10^ Department of Medical Oncology The Netherlands Cancer Institute, Antoni van Leeuwenhoek Amsterdam The Netherlands; ^11^ Department of Pathology University Medical Centre Utrecht The Netherlands; ^12^ Centre for Tumour Biology, Barts Cancer Institute, Queen Mary University of London London UK; ^13^ Faculty of Dentistry, Oral & Craniofacial Science King's College London London UK; ^14^ Head and Neck Pathology Guy's & St Thomas' NHS Foundation Trust London UK; ^15^ Department of Pathology GZA‐ZNA Hospitals Antwerp Belgium; ^16^ Division of Research Peter Mac Callum Cancer Centre Melbourne Australia; ^17^ Department of Pathology Tata Memorial Centre‐ACTREC, HBNI Mumbai India

**Keywords:** lymph node, triple negative breast cancer, digital pathology, computational pathology, germinal centre, sinus, deep learning

## Abstract

The suggestion that the systemic immune response in lymph nodes (LNs) conveys prognostic value for triple‐negative breast cancer (TNBC) patients has not previously been investigated in large cohorts. We used a deep learning (DL) framework to quantify morphological features in haematoxylin and eosin‐stained LNs on digitised whole slide images. From 345 breast cancer patients, 5,228 axillary LNs, cancer‐free and involved, were assessed. Generalisable multiscale DL frameworks were developed to capture and quantify germinal centres (GCs) and sinuses. Cox regression proportional hazard models tested the association between *smuLymphNet*‐captured GC and sinus quantifications and distant metastasis‐free survival (DMFS). *smuLymphNet* achieved a Dice coefficient of 0.86 and 0.74 for capturing GCs and sinuses, respectively, and was comparable to an interpathologist Dice coefficient of 0.66 (GC) and 0.60 (sinus). *smuLymphNet*‐captured sinuses were increased in LNs harbouring GCs (*p* < 0.001). *smuLymphNet*‐captured GCs retained clinical relevance in LN‐positive TNBC patients whose cancer‐free LNs had on average ≥2 GCs, had longer DMFS (hazard ratio [HR] = 0.28, *p* = 0.02) and extended GCs' prognostic value to LN‐negative TNBC patients (HR = 0.14, *p* = 0.002). Enlarged *smuLymphNet*‐captured sinuses in involved LNs were associated with superior DMFS in LN‐positive TNBC patients in a cohort from Guy's Hospital (multivariate HR = 0.39, *p* = 0.039) and with distant recurrence‐free survival in 95 LN‐positive TNBC patients of the Dutch‐N4plus trial (HR = 0.44, *p* = 0.024). Heuristic scoring of subcapsular sinuses in LNs of LN‐positive Tianjin TNBC patients (*n* = 85) cross‐validated the association of enlarged sinuses with shorter DMFS (involved LNs: HR = 0.33, *p* = 0.029 and cancer‐free LNs: HR = 0.21 *p* = 0.01). Morphological LN features reflective of cancer‐associated responses are robustly quantifiable by *smuLymphNet.* Our findings further strengthen the value of assessment of LN properties beyond the detection of metastatic deposits for prognostication of TNBC patients. © 2023 The Authors. *The Journal of Pathology* published by John Wiley & Sons Ltd on behalf of The Pathological Society of Great Britain and Ireland.

## Introduction

Solid tumours engage the lymphatic system, whereby the draining lymph nodes (LNs) are often the first site of dissemination outside the primary tumour [1]. For the treatment of invasive breast cancer, the tumour‐draining LNs, including the sentinel lymph node (SLN), are routinely excised, and details of the presence and size of LN metastases provide the basis for pathological staging. However, extensive dissection of all draining LNs does not reduce the mortality of breast cancer patients [2]. In recent years, surgical management of the axilla has become less radical, with fewer complete axillary clearances for those with small volume metastasis, for example of micrometastasis (≤2 mm). As demonstrated in the International Breast Cancer Study Group (IBCSG) trial 23‐01, eliminating axillary dissection had no adverse effect on survival compared to axillary dissection in early breast cancer patients with ≥1 micrometastasis [3].

Above and beyond the detection of metastasis, LNs serve as immunological hubs between the tumour and the patient's systemic immunity and provide an opportunity to study systemic host defence mechanisms, both pro‐ and anti‐tumoural, and their role in likely disease trajectories [1]. Such responses in the LNs include changes in fibroblastic architecture, the abundance of macrophages in sinuses, and hyperplasia of lymphoid follicles [4]. These morphological changes can be captured by computational approaches, such as deep learning (DL)‐based algorithms. A series of competitive international challenges have demonstrated the utility of exploring digitised whole‐slide images (WSI) of LNs, e.g. CAMELYON16 and 17, however, to date, with the focus only on cancer cell detection [5, 6]. By manually assessing ~3,000 H&E‐stained LNs and patient‐matched primary tumours on glass slides [7], we and others have demonstrated a higher risk of developing distant metastases, in particular in TNBC patients with low levels of tumour‐infiltrating lymphocytes (TILs), when the cancer‐free LNs lacked germinal centre (GC) formation [7, 8, 9, 10]. GCs are highly organised structures with an inner B‐cell follicle and an outer T‐cell zone that generate long‐lived memory B cells and plasma cells. Since these highly proliferative GC cells show some morphological similarities to cancer cells (e.g. size of cells), lymphoid follicle detection using convolutional neural networks (CNNs) has been proposed to exclude these areas for tumour detection in LNs [11]. However, DL‐based algorithms to capture the formation of GCs and other morphometric immune responses in LNs have, so far, not been utilised, in particular to determine whether their patterns hold clinically relevant information.

CNNs have superior performance in imaging tasks to alternative models, mainly due to their efficient parameter‐sharing between kernels and local connectivity properties [11, 12]. Based on the standard CNN framework, fully convolutional networks (FCNs) replace fully connected layers at the end of the network with convolutional layers, enabling pixel‐level classification and a low dimensional reconstruction of the input [12]. In biomedical applications, where datasets are often sparse relative to other computer vision tasks, the U‐Net architecture has proven to be an effective network for segmentation [13, 14]. However, whilst the standard U‐Net architecture is trained on single‐scale images, histopathologists analyse glass slides at multiple magnifications and integrate information from multiple fields of view when making clinical diagnostic, and prognostic factor, decisions. Thus, currently, this single‐scale feature encoding of images used by most convolutional models is not commensurate with a pathologist's visual assessment of multiple fields. In light of this, recent methods integrating a multiscale feature extractor into the U‐Net architecture, mimicking a histopathologist's assessment, have been developed and demonstrated superior segmentation performance on a range of medical image data modalities [15].

For this study, we built a supervised multiscale U‐Net‐based DL framework named *smuLymphNet* to capture and quantify GCs and sinuses within LNs on digitised H&E‐stained WSIs of 5,228 cancer‐free and involved LN sections from both LN‐negative and LN‐positive breast cancer patients, enriched for TNBC cases. We benchmarked *smuLymphNet* performance relative to manual LN assessments of four pathologists. By applying *smuLymphNet* to a retrospective breast cancer cohort and a clinical trial dataset, both with extensive longitudinal outcome data, we have revealed associations between *smuLymphNet*‐predicted immune features in LNs and the risk of subsequently developing distant metastasis in TNBC patients, which could be applied to tailor clinical therapy and to expand response assessment aspects in future clinical trials for these high‐risk patients.

## Materials and methods

Research ethics approval was obtained from the respective local research ethics committees (KHP Cancer Biobank REC reference 18/EE/0025, Barts Cancer Institute REC reference 21/EE/0072 until January 2026, Medical Ethics Committee of Tianjin Medical University Cancer Institute and Hospital, Ek2020021), and the institutional review board of the Netherlands Cancer Institute, clinical trial information: NCT03087409.

### Study population

**Figure 1:**
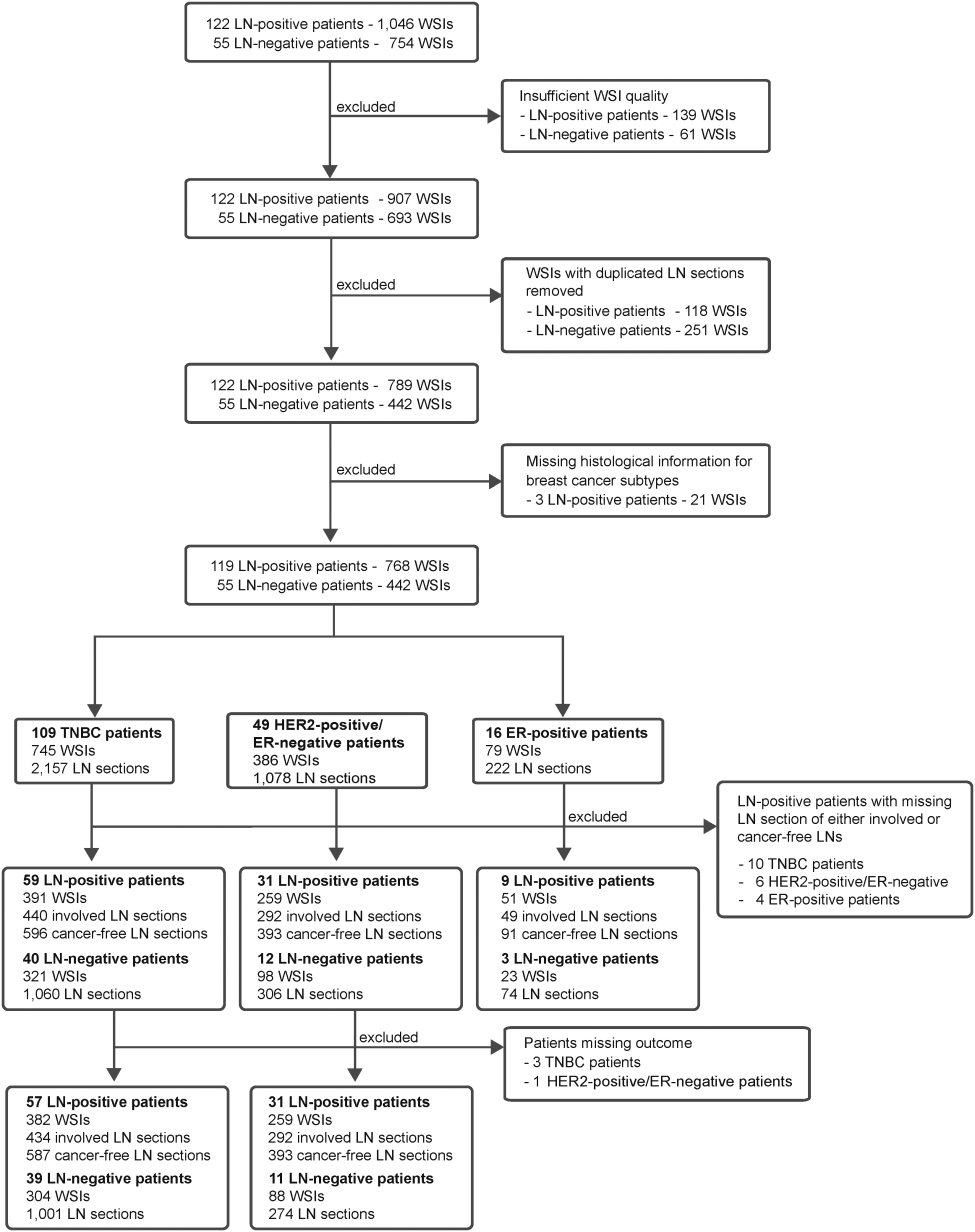
CONSORT diagram outlining data selection process. A retrospective dataset of 1,800 WSIs from 177 breast cancer patients from Guy's Hospital (London, UK) was retrieved. All WSIs were visually inspected, and WSIs with poor quality or pen marks were removed. Based on the LN section level classification obtained from the patient's histology report and after a visual assessment of all sections pertaining to a single LN, a single LN section level for each LN was selected. WSIs from three LN‐positive breast cancer patients were removed as the histological subtypes for these carcinomas were missing. The final dataset was split into three groups based on histological breast cancer subtype, namely in triple negative (TNBC), HER2‐positive/ER‐negative, and ER‐positive (HER2‐positive and HER2‐negative) breast cancer patients.

This is a retrospective study with a total of 345 patients across four independent cohorts. The main cohort consisted of 177 patients (122 LN‐positive [metastasis was reported in at least 1 LN] and 55 LN‐negative patients) with invasive breast carcinoma treated between 1984 and 2002 at Guy's Hospital in London, UK. An initial 1,800 H&E slides were scanned and digitised at magnification ×40 (0.23 μm/pixel). After manually assessing all WSIs, those of insufficient quality for analysis were removed (see Supplementary materials and methods for details). This resulted in 1,143 high‐quality WSIs of 3,301 LN sections from 154 breast cancer patients with extensive clinical‐pathological data (CONSORT diagram in Figure 1; see Supplementary materials and methods for details). We also obtained WSIs from five LNs of breast cancer patients from (1) Barts Cancer Institute (London, UK) (referred to as Barts); (2) Tianjin Medical University (Tianjin, PR China) (referred to as Tianjin), and (3) 180 LN sections from SLN biopsies from six Guy's Hospital breast cancer patients. A set of 174 WSIs of H&E‐stained LNs (114 /174 involved LNs) of 95 LN‐positive TNBC patients from the Dutch‐N4plus trial [16] was used as an external validation cohort (supplementary material, Table S1). We had previously assessed GC formation in 1,803 cancer‐free and involved H&E‐stained LNs from 161 LN‐positive hormone receptor‐negative breast cancer patients (Tianjin cohort) [17]. A pathologist (FL) manually assessed the subcapsular sinus (SCS) in LNs of 99 of the 161 TNBC Tianjin cohort. In 14 patients, LNs had insufficient tissue for subscapular measurement, resulting in a final cohort of 1,568 LNs from 85 LN‐positive TNBC patients (supplementary material, Table S2).

Patient selection and data analyses are reported according to Reporting Recommendations for Tumor Marker Prognostic Studies (REMARK) criteria [18].

### Study design

**Figure 2:**
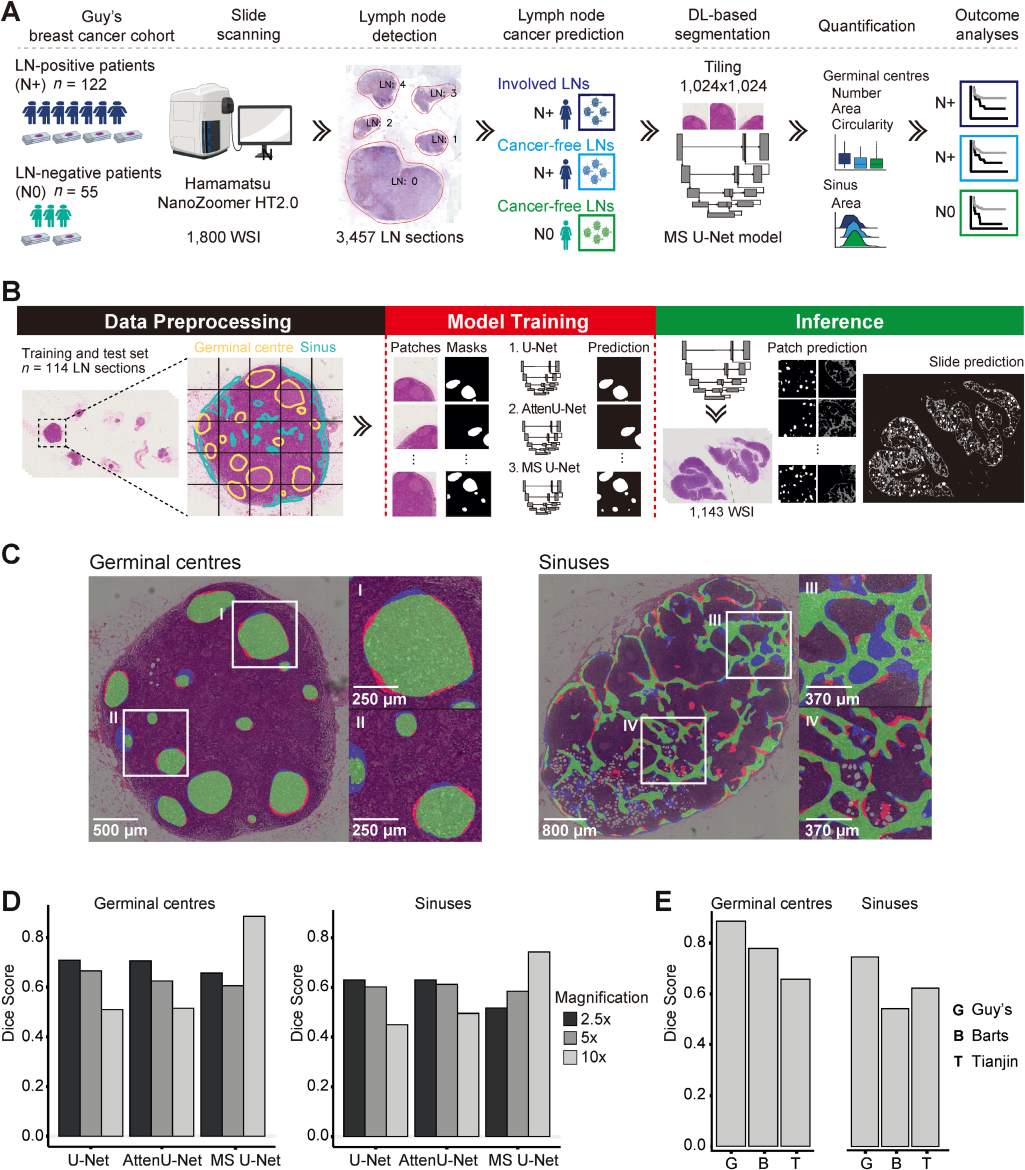
*smuLymphNet* framework to capture GCs and sinuses on H&E‐stained LN WSIs. (A) Schematic representation of implementation for *smuLymphNe*t framework: (1) digitising diagnostic LN glass slides from LN‐positive and LN‐negative patients diagnosed at Guy's Hospital; (2) LN‐detection algorithm to determine boundaries of each individual LN section on a WSI using an Otsu‐based thresholding method and contouring algorithm; (3) Neural Condition Random Field (NCRF) LN cancer metastasis prediction model to determine LN status for each LN section; (4) supervised deep learning pipeline using multiscale U‐Net model that assimilates semantic information from multiple resolutions to segment GCs and sinuses; (5) quantification of detected immune features, including number, mean area, and mean circularity of GCs per LN and total area of sinuses in LN normalised by LN area; (6) outcome analyses with immune features as variates. (B) Segmentation DL‐based pipeline is divided into data preprocessing, model training, and inference. In the data preprocessing step, LN sections were manually annotated for both GCs (marked in yellow) and sinuses (marked in blue) and split into tiles along with ground‐truth binary masks. Tiles and masks of 114 LN sections form the input to train and evaluate FCNs based on U‐Net architecture. Three models were evaluated: U‐Net, attention U‐Net (AttenU‐Net), and multiscale U‐Net (MS U‐Net). In the inference part, models were applied to 1,143 WSIs using the tile‐based approach, and predictions were stitched together to produce entire segmentation masks. (C) Two examples of MS U‐Net model segmentation. The MS U‐Net pixel‐wise prediction is overlaid on H&E image. True positives, i.e. GC or sinus pixels correctly predicted as GC or sinus, are shown in green; false positives, i.e. background pixels were incorrectly predicted as GC or sinus in red; and false negatives, i.e. GC or sinus pixels were incorrectly predicted as background in blue. (D) Bar plots show Dice coefficients of three CNN models at ×2.5, ×5, and ×10 magnification on Guy's cohort test set. GC prediction is shown on left, sinuses on right. (E) To test models' generalisability, bar plots display Dice coefficients for MS U‐Net on five LNs of breast cancer patients obtained from Guy's (G), Barts (B), and Tianjin (T) hospitals.

The *smuLymphNet* DL‐based framework (Figure 2A) to capture and quantify morphological perturbations in axillary LNs from breast cancer patients consists of (1) digitising the diagnostic LN glass slides (details of data collection are provided in supplementary methods); (2) a LN‐detection algorithm to determine the boundaries of each LN section on the WSI using an Otsu‐based thresholding [19] and contouring algorithm; (3) a LN metastasis classifier to determine involved or cancer‐free LNs [20]; (4) a supervised DL‐pipeline for the segmentation of GCs and sinuses (Figure 2B); and (5) the quantification of the number, size, and shape of the predicted features.

### DL framework

For the segmentation of GCs and sinuses in LNs, we tested three FCNs based on a U‐Net architecture with symmetrical encoder‐decoder paths: (1) a standard U‐Net architecture with five convolution blocks and skip connections (referred to as U‐Net), (2) a U‐Net model with an attention mechanism that upweights salient features during training (referred to as AttenU‐Net) [21], and (3) a multiscale U‐Net approach that assimilates semantic information from different scales during training using atrous convolutions [22] (referred to as MS U‐Net) (see Supplementary materials and methods and supplementary material, Figure S1). A single LN section was selected from 114 H&E‐stained WSIs from Guy's Hospital (London, UK) and manually annotated for GCs and sinuses by a pathologist (FL). Each annotated LN was divided into a series of overlapping equally sized tiles at magnifications of ×2.5, ×5, and ×10. Models were trained on tiles from 100 LN sections, with tiles from nine LN sections used for validation and tiles from five LN sections used as a holdout test set to evaluate model performance. Details for tile preprocessing and the model training procedures are provided in Supplementary materials and methods.

### Morphological feature quantification

For each LN, we captured the (1) number, (2) mean area, and (3) mean circularity of GCs (details of calculations are in Supplementary materials and methods). To estimate the overall area of the sinuses within a LN, the *smuLymphNet‐*captured sinuses were summed and normalised by overall LN size, due to a statistically significant correlation between the LN size and overall sinus area (supplementary material, Figure S2).

### Interpathologist concordance

A single LN on 24 WSIs was randomly selected for manual annotation by four pathologists (FL, ASh, SR, PG) using QuPath version 0.3.0. [23]. The ground‐truth binary masks of the 24 pathologist‐annotated LNs were compared for every pair of pathologists using the Dice coefficient [24].

### SCS quantification

A heuristic method was implemented to calculate the width of the SCS. Four points were selected based on a reference axis along the LN, chosen as the longest diameter across the LN section. Two points were determined as the intersection of the axis with the SCS on both sides and two at the intersection of the SCS based on a second axis orthogonal to the first. These four measurements were averaged to give the final indication of the SCS width (Equation 1) for each LN:

(1) $$\mathrm{SCS}\;\text{width}\;\mathrm{per}\;\text{node}=\frac{w_{1}+w_{2}+w_{3}+w_{4}}{4}$$

where w is the diameter at each of the selected points on the SCS.

### Statistical analyses

Standard summary statistics were calculated to establish associations between morphometric immune features and patients' prognosis. In the Guy's and Tianjin cohorts, the primary endpoints were distant metastasis‐free survival (DMFS), defined as the date of first invasive recurrence or second primary tumour or death from any cause. The endpoint in the Dutch‐N4Plus series was distant recurrence‐free survival (DRFS). Given the large number of LN sections per patient in the Guy's cohort, morphometric features were averaged across all assessed LNs for outcome analyses. In the Dutch‐N4Plus series, the total number of GCs across all assessed LNs and the LN with the maximum sinus area were used to assess outcome, as a much lower number of LNs was available per patient. We performed an iterative process to determine the optimal cut‐off points by a minimal *p* value approach [25]. Kaplan–Meier methods were used to compare survival curves across groups. Cox regression proportional hazards models were performed to estimate the hazard ratios according to clinicopathological and histologically assessed features across all endpoints in univariate and multivariate analyses. The statistical significance of features was assessed using the log‐likelihood ratio test across all cohorts, whereby a two‐sided *p* < 0.05 was considered significant. We used the statistical language R (version 4.1.1) to calculate the statistics [26].

## Results

### A multiscale embedded DL framework to capture immune responses in digitised LN WSIs

The DL framework *smuLymphNet* is illustrated schematically in Figure 2A. Amongst the three FCN models tested (Figure 2B, supplementary material, Figure S1), the MS U‐Net model performed the best at an input tile magnification of ×10 when features were learned at a combined magnifications of ×10, ×5, and ×2.5, with 0.86 (standard error [SE] = 0.04) and 0.74 (SE = 0.05) Dice coefficients for GC and sinus segmentation respectively. The highest Dice coefficients for the single‐scale U‐Net and AttenU‐Net models were 0.69 (SE = 0.17) and 0.62 (SE = 0.06) for GC and sinus segmentation, respectively, at an input tile magnification of ×2.5 (Figure 2C). Given that we have previously demonstrated the prognostic utility of GC number [7], we calculated an F1 score of predicted GC count and showed that the MS U‐Net model achieved 91%. To test the model's ability to generalise across various staining and acquisition protocols, we applied *smuLymphNet* to five LN WSIs obtained from two other hospitals (Barts, Tianjin). We observed that the Dice coefficients of GC segmentation were 0.78 (SE = 0.02) and 0.64 (SE = 0.09) for the Barts and Tianjin scanned LNs, respectively. For the *smuLymphNet‐captured* sinuses, the Dice coefficients decreased to an average of 0.55 (SE = 0.04) and 0.64 (SE = 0.05) for the Barts and Tianjin LNs, respectively (Figure 2D). Next, *smuLymphNet* was applied to digitised WSIs of LN sections from sentinel LN biopsies from six breast cancer patients diagnosed at Guy's Hospital, demonstrating its capability to capture GCs and sinuses on SLNs, now more commonly assessed in the current standard of care management of invasive breast cancer patients (supplementary material, Figure S3).

### Interpathologist concordance assessment of GCs and sinuses

**Figure 3:**
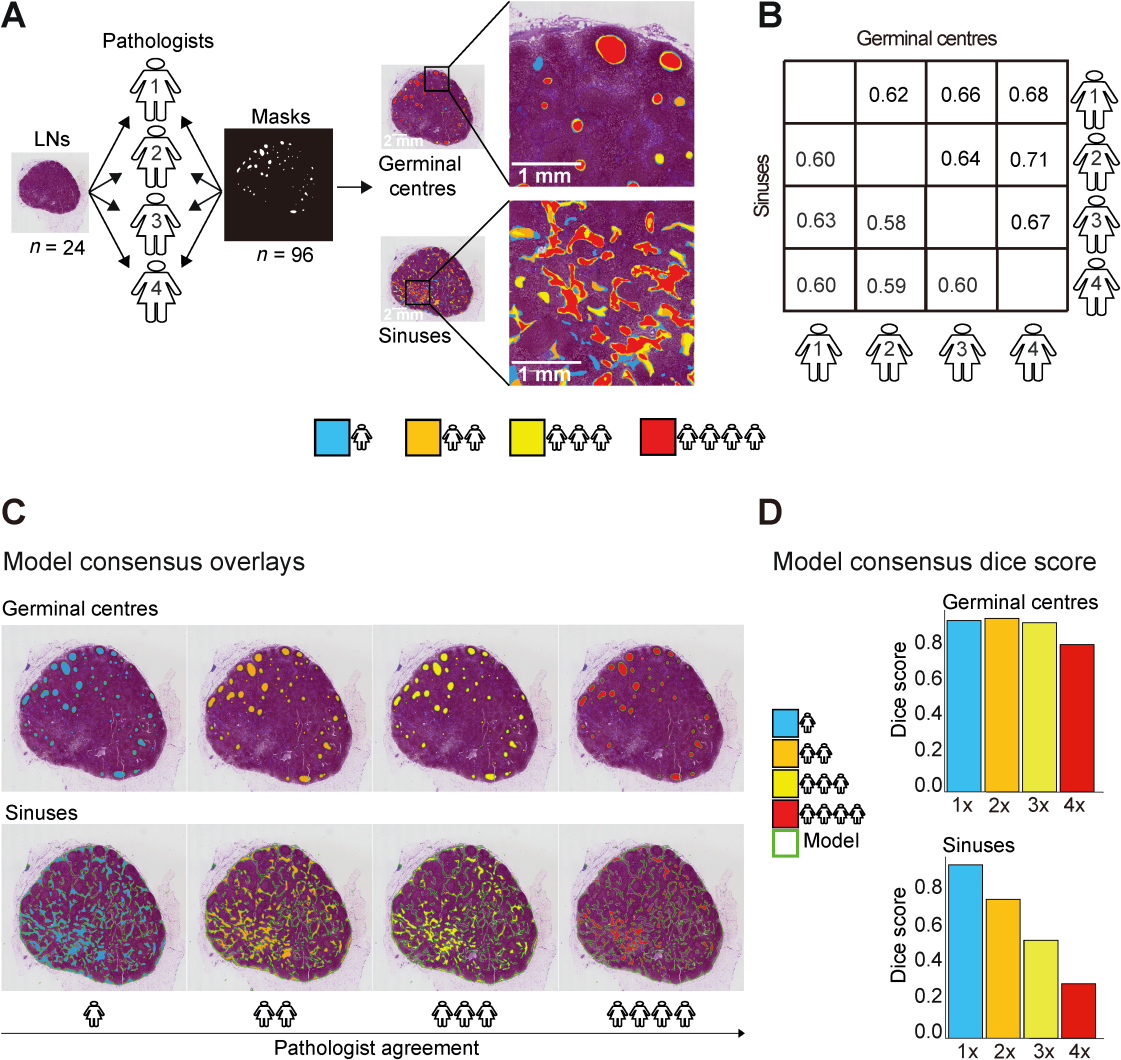
Interpathologist concordance. (A) Four pathologists annotated GCs and sinus areas of the same LNs on 24 WSIs using QuPath version 0.3.0. A binary mask was generated for each pathologist from the annotation files. Heatmaps illustrate the agreement between pathologists on a LN section as an example for GC (top) and sinus (bottom) annotation. The colour indicates how many pathologists marked a given area. Blue shows where only one pathologist annotated the area as a GC or sinus, and orange, yellow, and red show areas where two, three, or four pathologists agreed respectively. (B) Confusion matrix of Dice coefficient illustrates pairwise pathologists' agreements for either GCs or sinus annotation. (C) Four heatmaps of same LN; each heatmap shows areas marked by a different number of pathologists, referred to as pathologist consensus. The model predictions are overlaid on the LN. Pathologist consensus colour scheme is as follows: four pathologists (red), three pathologists (yellow), two pathologists (orange), and one pathologist (blue). Model prediction is highlighted by green contours. (D) Bar plot shows Dice coefficient for model predictions versus different sets of annotations derived from different levels of pathologist consensus (one to four pathologists agreeing) for the single LN seen in the heatmaps.

Pathologist annotations provide the gold standard to which DL models are compared. To contextualise *smuLymphNet's* performance, we determined the interpathologist variability in assessing GC and sinuses. For this purpose, four pathologists (FL, SR, PG, ASh) manually annotated 24 LNs (Figure 3A, detailed in Supplementary materials and methods). Based on pair‐wise Dice coefficients, the pathologists agreed at a slightly worse level than the *smuLymphNet* performance of GC annotation with mean Dice coefficients of 0.66 (SE = 0.013) for GC and 0.60 (SE = 0.006) for sinus annotation (Figure 3B–D), supporting the finding that our *smuLymphNet* framework can be utilised to analyse large WSI data.

### *smuLymphNet* ‐captured GCs show an association with disease progression

**Figure 4:**
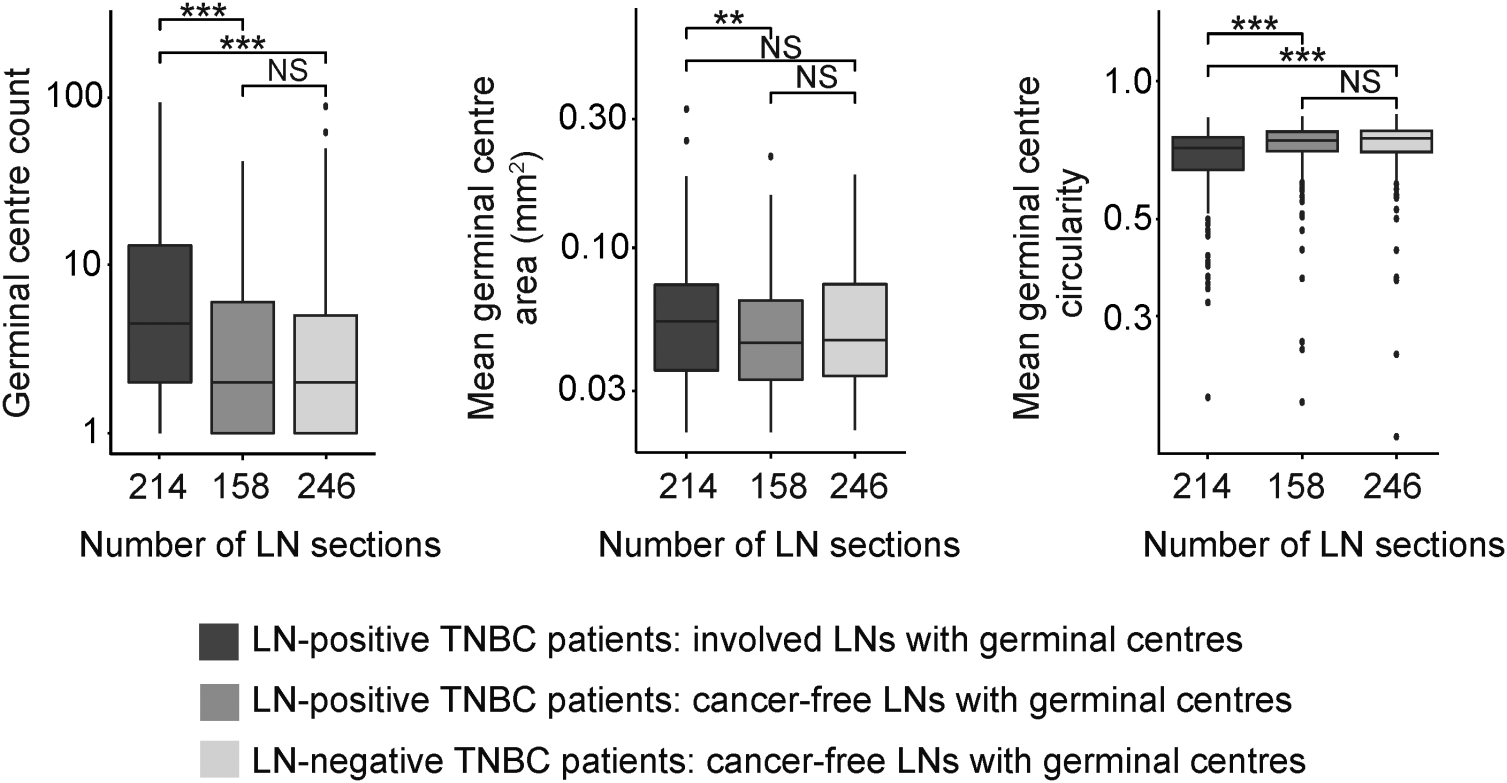
Properties of *smuLymphNet*‐captured GCs. The LN sections were separated into (1) involved LNs with GCs from LN‐positive TNBC patients, (2) cancer‐free LNs with GCs from LN‐positive TNBC patients, and (3) cancer‐free LNs with GCs from LN‐negative TNBC patients. Left to right: boxplots display distribution per LN section for GC count, mean GC area (mm^2^), and mean GC circularity. Statistical significance was assessed using a two‐sided Wilcoxon rank sum test (**p ≤ 0.01, ***p ≤ 0.001, NS = not significant).

Next, the *smuLymphNet* framework was applied to 1,143 WSIs of H&E‐stained LN sections from 154 breast cancer patients, encompassing (1) 2,096 LNs from 99 TNBC patients, (2) 991 LNs from 43 human epidermal growth factor receptor 2 (HER2)‐positive/oestrogen receptor (ER)‐negative patients, and (3) 214 LNs from 12 ER‐positive patients (see CONSORT diagram in Figure 1). The number of *smuLymphNet*‐captured GCs per LN was independent of all assessed LNs per patient (supplementary material, Figure S4). For TNBC patients, LNs had a mean GC count of 2 (range, 0–92), a mean area of 0.06 mm^2^ (range, 0.022–0.34 mm^2^), and a mean circularity of 0.75 (range, 0.17–0.9). In HER2‐positive/ER‐negative and ER‐positive breast cancer patients, the mean GC area per LN of 0.05 mm^2^ (range, 0.022–0.24 mm^2^) and the mean GC circularity of 0.75 and 0.76 (range, 0.22–0.9 and 0.34–0.87) were comparable; however, only one GC per LN (range, 0–78 and 0–12) was on average observed in LNs (supplementary material, Figure S5A,B). In TNBC patients, the involved LNs had, on average, four GCs, in contrast to cancer‐free LNs (from both LN‐positive and LN‐negative TNBC patients), where only one GC was found (range, 0–92, 0–39 and 0–87, Wilcoxon rank sum test, *p* < 0.001, Figure 4). Involved LNs displayed GCs with larger mean areas of 0.065 mm^2^ compared to 0.056 mm^2^ cancer‐free LNs in LN‐positive patients (Wilcoxon rank sum test, *p* < 0.01, Figure 4), but this did not differ significantly from the mean area in cancer‐free LNs of LN‐negative patients. GC area was highly correlated with mean GC numbers (Pearson's *r* = 0.83, *p* < 0.001, data not shown). The GC circularity was highest in cancer‐free LNs of LN‐negative TNBC, followed by cancer‐free and then involved LNs of LN‐positive TNBC (Wilcoxon rank sum test, *p* < 0.001, Figure 4). Overall, involved LNs had, on average, more GCs, with larger surface areas and more irregularities than cancer‐free LNs.

Having previously shown that manual assessment of GCs in LNs carries prognostic value in LN‐positive TNBC [7], we asked whether *smuLymphNet*‐quantified GCs held prognostic value for these high‐risk patients. In the Guy's cohort, LN‐positive TNBC patients had longer DMFS when their involved and cancer‐free LNs displayed on average ≥2 GCs across all assessed (for involved LNs, HR = 0.45, 95% CI: 0.23–0.95, *p =* 0.04; and for cancer‐free LNs HR = 0.28, 95% CI: 0.09–0.94, *p =* 0.02; Table 1 and supplementary material, Figure S5C). A similar association was observed in LN‐negative TNBC patients (cancer‐free LNs HR = 0.14, 95% CI: 0.03–0.63, *p =* 0.002; Table 1 and supplementary material, Figure S5C). In multivariate models, when adjusted for age at diagnosis, histological grade, and number of involved LNs, the binary cut‐off for GCs in cancer‐free LNs remained statistically associated with DMFS in LN‐negative TNBC (HR = 0.2, 95% CI: 0.04–0.95, *p* = 0.04, Table 1). An increased mean GC area in LNs was associated with superior prognostic value in involved LNs of LN‐positive TNBC patients (univariate HR = 0.44, 95% CI: 0.21–0.92, *p* = 0.03, and adjusted for age at diagnosis, histological grade, and number of involved LNs HR = 0.42, 95% CI: 0.19–0.94, *p* = 0.04, Table 1), and in cancer‐free LNs of LN‐negative TNBC patients (univariate HR = 0.22, 95% CI: 0.05–0.98, *p* = 0.02, Table 1; supplementary material, Figure S5C). When assessing GC circularity, regular GC formation in involved LNs of LN‐positive TNBC patients was associated with a superior prognosis (univariate HR = 0.34, 95% CI: 0.13–0.85, *p* = 0.03, and adjusted for age at diagnosis, histological grade, and number of involved LNs HR = 0.26, 95% CI: 0.08–0.86, *p* = 0.03; Table 1). Taken together, patients with LNs harbouring fewer GCs, and as such smaller areas and of irregular shapes, had a higher risk of developing distant metastases.

**Table 1:** Univariate and multivariate Cox proportional hazard analyses of germinal centre properties in LNs of patients from Guy's TNBC cohort.

Guy's TNBC cohort	Model *P*	Hazard ratio	95% CI
**Univariate distant metastasis free survival**			
LN‐positive TNBC (*N* = 57), involved LNs			
Mean GC number per patient (<2 versus ≥2)	0.04	0.454	0.23–0.95
Mean GC area per patient (≤0.015 mm^2^ versus >0.015 mm^2^)	0.03	0.441	0.21–0.92
Mean GC circularity per patient (≤0.69 versus >0.69)	0.03	0.335	0.13–0.85
LN‐positive TNBC (*N* = 57), cancer‐free LNs			
Mean GC number per patient (<2 versus ≥2)	0.02	0.283	0.09–0.94
Mean GC area per patient (≤0.015 mm^2^ versus >0.015 mm^2^)	0.1	0.496	0.20–1.22
Mean GC circularity per patient (≤0.69 versus >0.69)	0.3	0.534	0.18–1.62
LN‐negative TNBC (*N* = 39), cancer‐free LNs			
Mean GC number per patient (<2 versus ≥2)	0.002	0.14	0.03–0.63
Mean GC area per patient (≤0.015 mm^2^ versus >0.015 mm^2^)	0.02	0.22	0.05–0.98
Mean GC circularity per patient (≤0.69 versus >0.69)	NA	NA	NA

	Covariate *P*	Model *P*	Hazard ratio	95% CI
**Multivariate distant metastasis‐free survival**				
Adjusted for age at diagnosis, histological grade, and number of involved				
LN‐positive TNBC (*N* = 57), involved LNs				
Mean GC number per patient (<2 versus ≥2)	0.05	0.03	0.431	0.18–1.01
Mean GC area per patient (≤0.015 mm^2^ versus >0.015 mm^2^)	0.04	0.02	0.422	0.19–0.94
Mean GC circularity per patient (≤0.69 versus >0.69)	0.03	0.04	0.262	0.08–0.86
LN‐positive TNBC (*N* = 57), cancer‐free LNs				
Mean GC number per patient (<2 versus ≥2)	0.08	0.03	0.329	0.09–1.15
Mean GC area per patient (≤0.015 mm^2^ versus >0.015 mm^2^)	0.13	0.05	0.485	0.19–1.25
Mean GC circularity per patient (≤0.69 versus >0.69)	0.33	0.2	0.520	0.14–1.92
Adjusted for age at diagnosis, histological grade				
LN‐negative TNBC (*N* = 39), cancer‐free LNs				
Mean GC number per patient (<2 versus ≥2)	0.04	0.06	0.20	0.04–0.95
Mean GC area per patient (≤0.015 mm^2^ versus >0.015 mm^2^)	0.19	0.2	0.352	0.07–1.66
Mean GC circularity per patient (≤0.69 versus >0.69)	NA	NA	NA	NA

Statistical significance was assessed using likelihood ratio tests.

To reduce the risk of biased performance estimation [27] of our *smuLymphNet* methodology, we next evaluated its performance in an external cohort of 174 involved and cancer‐free LNs from 95 LN‐positive TNBC patients of the Dutch‐N4plus trial [28]. In this trial, breast cancer patients with at least four involved LNs but without distant metastases at diagnosis had been randomised to conventional 5‐fluorouracil‐epirubicin‐cyclophosphamide (FEC) chemotherapy or the same therapy but whose last course was replaced by high‐dose cyclophosphamide‐thiotepa‐carboplatin (CTC) chemotherapy with autologous stem cell support. Although only 18/95 (19%) TNBC patients of the Dutch‐N4plus trial had >2 GCs in all of their patients' available LNs, these had a superior DRFS; however, due to the limited cohort size, this did not reach statistical significance (HR = 0.52, 95% CI: 0.24–1.13, *p* = 0.097; supplementary material, Table S3A and Figure S5D).

### Increased *smuLymphNet* ‐captured sinus areas are present in LNs of patients with longer time to distant recurrence

**Figure 5:**
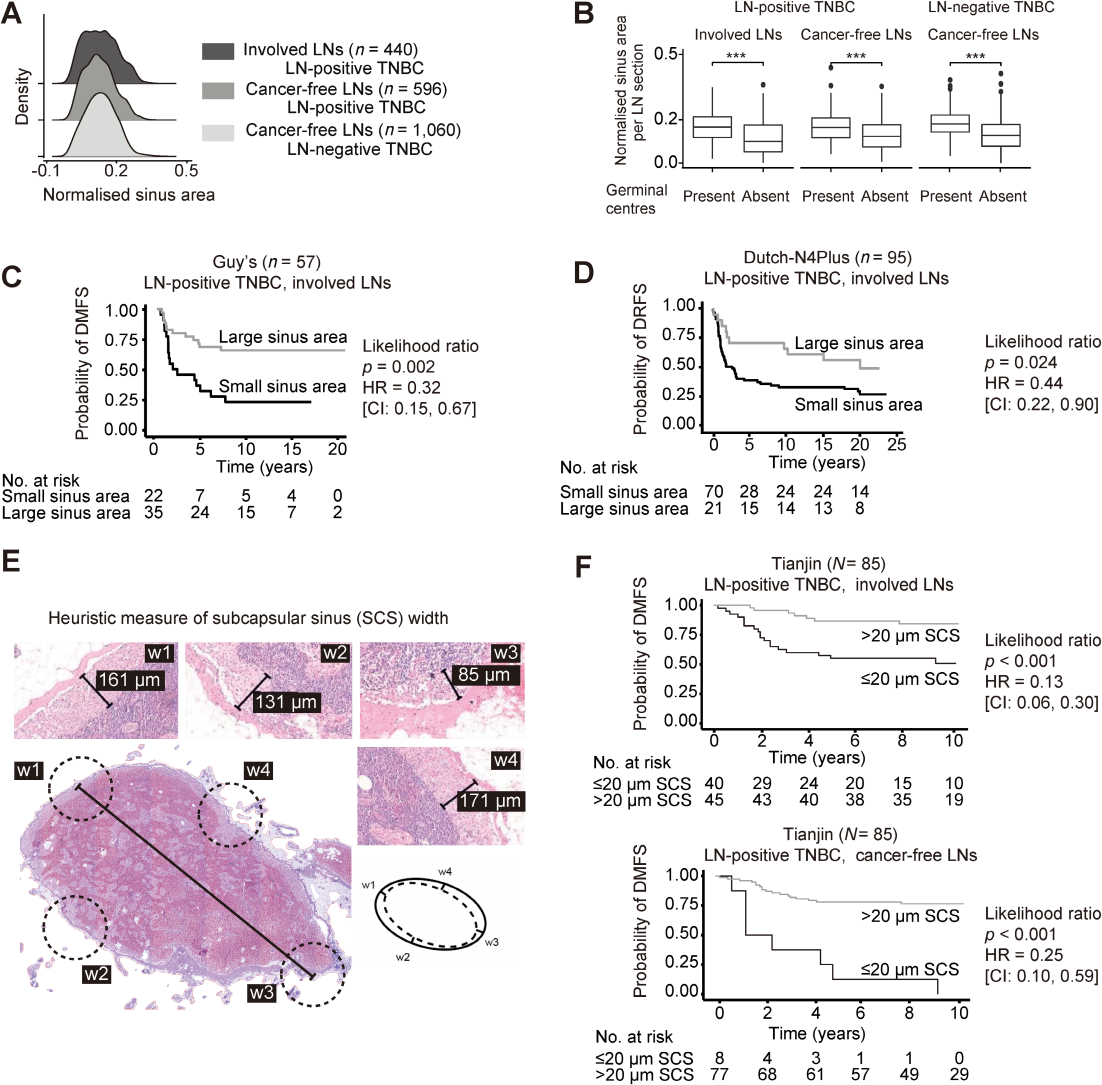
*smuLymphNet*‐captured sinuses, their quantification, and association with disease progression. (A) The LN sections were separated into (1) involved LNs, (2) cancer‐free LNs from LN‐positive TNBC patients, and (3) cancer‐free LNs from LN‐negative TNBC patients. Normalised sinus areas per LN were determined by calculating the total *smuLymphNet*‐based sinus area divided by the LN section area. Density plots show distribution of normalised sinus area per LN section. Statistical significance was assessed by false discovery rate (FDR)‐adjusted Kruskal–Wallis tests. (B) Boxplots showing normalised sinus area per LN section separated by LNs in which GCs were present or absent. Statistical significance was assessed using a two‐sided Wilcoxon rank sum test (***p < 0.001). (C) Kaplan–Meier analyses of distant metastasis‐free survival (DMFS) for LN‐positive TNBC patients of Guy's cohort. (D) Kaplan–Meier curve of distant recurrence‐free survival (DRFS) for TNBC patients of Dutch‐N4plus trial. Patients for both cohorts were dichotomised based on their normalised sinus area of LNs. *P* values correspond to likelihood ratio tests. Hazard ratio (HR) and 95% confidence intervals (95% CI) are listed. (E) To capture the SCS area by visually assessing H&E‐stained LN section. A heuristic measure of SCS width was implemented by assessing SCS at four points in a LN, and their average resulted in the final SCS width. (F) Kaplan–Meier curves of DMFS for LN‐positive TNBC patients of Tianjin cohort. Patients were dichotomised based on their SCS width in all assessed involved or cancer‐free LNs as categorical variables. *P* values correspond to likelihood ratio tests. HR and 95% CIs are listed.

Utilising the *smuLymphNet* framework, we assessed the sinus areas in cancer‐free and involved LNs. Amongst all assessed LNs of TNBC patients, the normalised sinus area was on average 0.14 mm^2^ (range, 0–0.41 mm^2^) (Figure 5A); however, this increased significantly when LNs displayed any GC formation (Figure 5B, Wilcoxon rank sum test, false discovery rate‐adjusted *p* < 0.001). In HER2‐positive/ER‐negative and ER‐positive breast cancer patients, normalised sinus areas were more variable, which may be inflated due to the small cohort sizes (supplementary material, Figure S6A,B). Nevertheless, LNs with GCs displayed larger normalised sinus areas (supplementary material, Figure S6C).

Next, we tested whether a normalised sinus area across all assessed LNs was associated with prognosis in TNBC patients. As shown in Figure 5C, in the Guy's cohort LN‐positive TNBC patients with involved LNs with normalised sinus area >0.13 mm^2^ had a better DMFS in univariate analyses (HR = 0.32; 95% CI: 0.15–0.67, *p* = 0.002; Table 2A; the optimal cut‐off curves are shown in supplementary material, Figure S7). In multivariate models, when adjusted for mean GC count, age at diagnosis, histological grade, and number of involved LNs, this binary cut‐off for sinus area in involved LNs remained statistically associated with DMFS (HR = 0.391; 95% CI: 0.16–0.95; *p* = 0.039, Table 2A). In the Dutch‐N4plus trial, external validation cohort, TNBC patients with LNs displaying *smuLymphNet*‐quantified sinus area greater than 0.13 mm^2^ had an overall superior DRFS in univariate analyses (HR = 0.44; 95% CI: 0.22–0.90, *p* = 0.024; supplementary material, Table S3B, Figure 5D) and added prognostic value to stromal TILs in multivariate analyses (HR = 0.50, CI: 0.25–1.02, covariate *p* = 0.056 and likelihood *p* = 0.043; supplementary material, Table S3B). Of note, the predefined cut‐off of 0.13 mm^2^ sinus area derived originally from LNs of the Guy's cohort was used for sinus area assessment in the LNs of the Dutch‐N4plus trial.

**Table 2:** Univariate and multivariate Cox proportional hazard analyses of sinus area in LNs of TNBC patients.

A. Guy's TNBC cohort	Covariate *P*	Model *P*	Hazard ratio	95% CI
**Univariate distant metastasis‐free survival**				
LN‐positive TNBC (*N* = 57), involved LNs				
Normalised sinus area (≤0.13 mm^2^ versus >0.13 mm^2^)		0.002	0.318	0.15–0.67
LN‐positive TNBC (*N* = 57), cancer‐free LNs				
Normalised sinus area (≤0.13 mm^2^ versus >0.13 mm^2^)		0.5	0.793	0.38–1.64
LN‐negative TNBC (*N* = 39), cancer‐free LNs				
Normalised sinus area (≤0.13 mm^2^ versus >0.13 mm^2^)		**0.7**	**0.802**	0.27–2.35
**Multivariate distant metastasis‐free survival**				
LN‐positive TNBC (*N* = 57), involved LNs				
Normalised sinus area (≤ 0.13 mm^2^ versus >0.13 mm^2^)	0.008	0.003	0.357	0.17–0.77
LN‐positive TNBC (*N* = 57), cancer‐free LNs				
Normalised sinus area (≤0.13 mm^2^ versus >0.13 mm^2^)	0.063	0.05	0.837	0.40–1.74
LN‐negative TNBC (*N* = 39), cancer‐free LNs				
Normalised sinus area (≤0.13 mm^2^ versus >0.13 mm^2^)	0.781	0.007	1.167	0.39–3.46
**Multivariate distant metastasis‐free survival**				
Adjusted for mean GC count, age at diagnosis, histological grade, and involved LN number				
LN‐positive TNBC (*N* = 57), involved LNs				
Normalised sinus area (≤0.13 mm^2^ versus >0.13 mm^2^)	0.039	0.01	0.391	0.16–0.95
LN‐positive TNBC (*N* = 57), cancer‐free LNs				
Normalised sinus area (≤0.13 mm^2^ versus >0.13 mm^2^)	0.528	0.05	0.752	0.31–1.83
Adjusted for mean GC count, age at diagnosis, and histological grade				
LN‐negative TNBC (*N* = 39), cancer‐free LNs				
Normalised sinus area (≤0.13 mm^2^ versus >0.13 mm^2^)	0.681	0.1	1.349	0.32–5.63

B. Tianjin TNBC cohort	Covariate *P*	Model *P*	Hazard ratio	95% CI
**Univariate distant metastasis‐free survival**				
LN‐positive TNBC (*N* = 85), involved LNs				
SCS width (≤ 20 μm versus >20 μm)		<0.001	0.130	0.06–0.30
LN‐positive TNBC (*N* = 85), cancer‐free LNs				
SCS width (≤20 μm versus >20 μm)		<0.001	0.246	0.10–0.59
**Multivariate distant metastasis‐free survival**				
Adjusted for total GC count				
LN‐positive TNBC (*N* = 85), involved LNs				
SCS width (≤20 μm versus >20 μm)	<0.001	<0.001	0.241	0.10–0.57
LN‐positive TNBC (*N* = 85), cancer‐free LNs				
SCS width (≤20 μm versus >20 μm)	<0.001	<0.001	0.201	0.08–0.50
**Multivariate distant metastasis‐free survival**				
Adjusted for total GC count, age, pTstage, pNstage, LVI, sTILs, and TILs				
LN‐positive TNBC (*N* = 85), involved LNs				
SCS width (≤20 μm versus >20 μm)	0.029	<0.001	0.334	0.13–0.89
LN‐positive TNBC (*N* = 85), cancer‐free LNs				
SCS width (≤20 μm versus >20 μm)	0.010	<0.001	0.211	0.06–0.69

Statistical significance was assessed using likelihood ratio test.

### Pathologists' assessment validates prognostic value of sinus area

To orthogonally validate the prognostic value of *smuLymphNet*‐captured sinuses, the width of the SCS, as a surrogate of the sinus area, was manually assessed by a pathologist in an independent set of LN‐positive TNBC patients from the previously examined Tianjin cohort [7]. SCS located beneath the LN capsule reflects on the overall LN conduit activities. A width heuristic was calculated using four positions of the LN to establish an average SCS width for each LN (Figure 5E). The statistics of the manual assessment of SCS are shown in supplementary material, Table S2, and detailed in the methods. An increased SCS width of ≥20 μm across all the assessed LNs was associated with a superior prognosis (Table 2B, Figure 5F). In multivariate models, when adjusted for total GC count, patient age, pathological tumour size (pT), LN stage (pN), the presence of lymphovascular invasion and of tertiary lymphoid structure, and stromal tumour‐infiltrating lymphocytes, which had been shown to be associated with DMFS in this cohort [7], the binary cut‐off for SCS widths remained statistically associated with DMFS in involved LNs (HR = 0.33, 95% CI: 0.13–0.89, *p* = 0.029, Table 2B) and cancer‐free LNs (HR = 0.21, 95% CI: 0.06–0.69, *p* = 0.01, Table 2B).

## Discussion

In this retrospective study, we developed a fully supervised DL framework, *smuLymphNet*, demonstrating that a multiscale U‐Net architecture could robustly capture morphological immune structures from digitised images of routine H&E‐stained slides from both axillary and sentinel LNs with high accuracy comparable to interpathological assessments. In alignment with our published studies [7, 8], our *smuLymphNet* framework recapitulated the finding of the prognostic value of the assessment of GC formation in LN‐positive TNBC and has now extended this association to LN‐negative TNBC patients. We revealed, for the first time, the prognostic significance of the morphological assessment of intranodal lymphatic sinuses in involved LNs in two independent TNBC cohorts, both by our DL‐based methodology and by manual assessment. Lastly, we demonstrated that these morphological features in LNs added prognostic value in a clinical trial TNBC cohort. This underscores the significant clinical potential of the relevance of immune responses reflected by LN morphology.

During cancer‐induced immune responses, LNs enlarge and remodel, featuring GC formation and growth of lymphatic sinuses (lymphangiogenesis), even before metastatic deposits are detected [7, 29]. In our study, both increased total sinus surface area and SCS width in LNs were associated with a better prognosis. A layer of CD169+ macrophages lines the SCS and is strategically positioned at the lymph–tissue interface to capture pathogens as they enter the LN [30]. This impedes the systemic dissemination of pathogens [31] and allows the presentation of intact antigens to B cells that reside directly underneath the SCS macrophage layer for the initiation of humoral immune responses and, in turn, to initiate GC formation. A high density of CD169+ macrophages in the LN sinus has been shown to be predictive of better clinical prognosis in some tumours [32]. During tumour progression, SCS macrophages become depleted and dissociate from the SCS [33]. Consequently, the width of the SCS narrows, in alignment with our observation that the SCS width was narrower in involved compared to cancer‐free LNs. Exploring the lymphatic sinus further with computational pathology approaches could provide additional clinically relevant diagnostic information and complement micro‐CT‐guided lymphangiography in breast and other cancers.

Segmentation of substructure‐specific morphological properties, such as sinuses, from H&E‐stained images is challenging for artificial intelligence (AI)‐based methods. The multiscale CNNs are trained by integrating surrounding context, morphology, and cellular information originating from different magnification levels in the learned feature representation of the network [34]. We showed that the multiscale architecture outperformed the single‐magnification FCNs for the segmentation task and mimics the actual assessment process applied by pathologists by integrating information from different scales. Despite this improved performance, a small number of necrotic and cancerous regions still led to false positive predictions of GCs, largely due to the computational resource trade‐off between image resolution and image size, limiting the amount of cell and nuclear semantics included in the learned model latent space. Another key challenge in computational pathology, which has slowed the adoption of this new paradigm in a clinical context, is the curation of large‐scale datasets that capture the inherent technical variability of WSIs from multiple institutes. This is particularly challenging in the face of supervised methods where the necessity of obtaining detailed annotated data for the development and validation of neural networks is not feasible [25]. As such, methods that use weak supervision to obviate the laborious task of obtaining detailed pathologists' annotations may provide a more efficient way to train these models on large scale datasets. To evaluate our model's performance fairly, we included the subjectivity of four pathologists' manual assessments and observed moderate interobserver agreement to recognise these polymorphic substructures, illustrating the difficulty of defining the ground truth for such tasks. Potentially, methods for generating crowd‐sourced noisy labels at scale and more sophisticated machine learning techniques to leverage them may provide new opportunities [35].

Since the detection of LN metastasis is critical for the diagnosis and staging of many solid tumours, pathology modernisation programmes in hospitals have started to evaluate AI‐based software tools with the aim of supporting pathologists' workload and improving the speed of assessment of microscopic examination. In the era of immunotherapy as a treatment choice for TNBC [36], the LN can potentially be used as an observation window for the patient's systemic immune responses of prognostic value [7, 8]. Currently, computational assessment of TIL counts is proposed as a prognostic biomarker for TNBC [37] by themselves or integrated into nomograms with established prognostic variables [38] through a public Grand Challenge organised by the International Immuno‐Oncology Biomarker Working Group (www.tilsinbreastcancer.org). Although we have demonstrated the generalisation of our models and have orthogonally validated our observations in an external cohort, further large‐scale validation of these findings, ideally in clinical trials in neoadjuvant and adjuvant settings, potentially facilitated at scale by federated or swarm‐learning‐based approaches [6, 39, 40], would be invaluable.

## Conclusions

Multiscale DL approaches are well suited to capture and quantify cancer‐associated alterations in axillary LNs on digitised WSIs. By assessing LNs above and beyond the presence and size of cancer cell deposits, our end‐to‐end *smuLymphNet* framework provides a tool to advance TNBC patient stratification, management, and prognostication and has the potential to benefit clinical practice.

## Author contributions statement

GV and ML were responsible for conceptualization, methodology, software, validation, formal analysis, investigation, data curation, original draft preparation, review and editing and visualisation. FL was responsible for conceptualization, methodology, validation, formal analysis, investigation, data curation, review and editing and visualisation. AL, NCK, SM and PG were responsible for methodology, software, validation, investigation and review and editing. ASh was responsible for validation and review and editing. AO and ST were responsible for data curation. TC, MO and SL were responsible for validation, review and editing and visualisation. CG was responsible for data curation review and editing. EA and TH were responsible for review and editing and visualisation. SJ and JLJ were responsible for review and editing. RS was responsible for methodology, data curation and review and editing. SEP and SR were responsible for review and editing. AS was responsible for conceptualization, methodology, software, validation, formal analysis, investigation, data curation, review and editing, visualisation, and funding acquisition. AG was responsible for conceptualization, methodology, software, validation, formal analysis, investigation, data curation, original draft preparation, review and editing, visualisation, and funding acquisition.

## Supporting information


Supplementary materials and methods
**Figure S1.** *smuLymphNet* pipeline and network architecture for FCN models
**Figure S2.** Correlation between sinus area and total LN area
**Figure S3.** *smuLymphNet*‐captured germinal centre and sinuses, their quantification in sentinel LNs
**Figure S4.** Germinal centre counts per patient is independent of number of LNs assessed
**Figure S5.** Germinal centre quantitative assessment and outcome analyses
**Figure S6.** Sinus area, quantitative assessment, and outcome analyses
**Figure S7.** Defining the prognostic sinus area cut‐off
**Table S1.** Clinical characteristics of patients from Dutch‐N4Plus TNBC cohort (*n* = 95)
**Table S2.** Clinical characteristics and immune features in LNs of patients from Tianjin TNBC cohort
**Table S3.** Univariate and multivariate Cox proportional hazard analyses in Dutch‐N4Plus TNBC cohort

## Data Availability

WSIs of H&E‐stained LNs were generated using NanoZoomer 2.0‐HT scanners (Hamamatsu Photonics UK, Ltd, Welwyn Garden City, UK) at magnification ×40. The resulting WSIs of the H&E‐stained axillary LNs from Guy's Hospital patients are available at Amazon Web Services (AWS) open data (https://registry.opendata.aws/guys-breast-cancer-lymph-nodes/). Code for the trained models, *smuLymphNet* inference and quantification framework, and related documentation are available at https://github.com/cancerbioinformatics/smuLymphNet.
