# Supplementary material for: Multiscale deep learning framework captures systemic immune features in lymph nodes predictive of triple negative breast cancer outcome in large‐scale studies
Source: J Pathol. 2023 May 25;260(4):376–89. doi: 10.1002/path.6088 (PMC10720675; doi:10.1002/path.6088)
Supplement: Supplementary file 1 — Supplementary materials and methods Figure S1. smuLymphNet pipeline and network architecture for FCN models Figure S2. Correlation between sinus area and total LN area Figure S3. smuLymphNet‐captured germinal centre and sinuses, their quantification in sentinel LNs Figure S4. Germinal centre counts per patient is independent of number of LNs assessed Figure S5. Germinal centre quantitative assessment and outcome analyses Figure S6. Sinus area, quantitative assessment, and outcome analyses Figure S7. Defining the prognostic sinus area cut‐off Table S1. Clinical characteristics of patients from Dutch‐N4Plus TNBC cohort (n = 95) Table S2. Clinical characteristics and immune features in LNs of patients from Tianjin TNBC cohort Table S3. Univariate and multivariate Cox proportional hazard analyses in Dutch‐N4Plus TNBC cohort [file PATH-260-376-s001.docx]

**Multiscale deep learning framework captures systemic immune features in lymph nodes predictive of triple negative breast cancer outcome in large-scale studies.**

G Verghese, M Li, F Liu *et al. J Pathol* <https://doi.org/10.1002/path.6088>

**Supplementary materials and methods**

**Supplementary Figures S1–S7**

**Supplementary Tables S1–S3**

**Supplementary materials and methods**

Reference numbers refer to the main text list

**Data collection and preparation**

This was a retrospective study with a total of 345 patients across four independent cohorts. The main cohort consisted of 177 patients (122 LN-positive and 55 LN-negative patients) with invasive breast carcinoma treated between 1984 and 2002 at Guy’s Hospital London, UK. An initial 1,800 H&E slides were scanned and digitised at magnification ×40 (0.23 µm/pixel) NanoZoomer 2.0-HT (Hamamatsu Photonics UK, Welwyn Garden City, UK). Whole-slide images (WSIs) were removed if they were of (i) insufficient quality (*n* = 200); (ii) contained the same LNs but from different levels (*n* = 369); (iii) patient-matched histopathological tumour information was missing (*n* = 21); and (iv) when a LN-positive breast cancer patient had WSI only from involved or only from cancer-free LNs (*n* = 20). This resulted in 1,143 high-quality WSIs of 3,301 LN sections from 154 breast cancer patients with extensive clinical-pathological data (CONSORT diagram in Figure 1). We also obtained WSIs of five LNs from (i) breast cancer patients from Barts Cancer Institute (London, UK) (referred to as Barts), digitised on a NanoZoomer S60 (Hamamatsu); and (ii) the Tianjin Medical University (Tianjin, PR China) (referred to as Tianjin), digitised on NanoZoomer 2.0-HT (Hamamatsu Photonics UK) as well as an additional 180 sentinel LN sections from six breast cancer patients from Guy’s Hospital digitised on a NanoZoomer S60 (Hamamatsu Photonics UK). A set of 174 H&E-stained LNs (114 /174 involved LNs), digitised on a Pannoramic 1000 (3DHistech, Budapest, Hungary), from 95 LN-positive TNBC patients from the Dutch-N4plus trial were used as an external validation cohort for the outcome analyses (supplementary material, Table S1). An orthogonal manual validation of the prognostic value of sinuses was performed using a cohort we previously assessed for GC formation in H&E-stained LNs from 161 hormone receptor-negative LN-positive breast cancer patients (Tianjin cohort, digitised on a NanoZoomer 2.0-HT (Hamamatsu Photonics UK) [17]. A subset of 99 LN-positive TNBC Tianjin patients (supplementary material, Table S2), including 1,803 cancer-free and involved LNs, were manually assessed by a pathologist (FL) for subcapsular sinus. Due to insufficient tissue quality, 14 patients were removed, resulting in a total of 85 patients and 1,568 LNs used for further analyses. Patient selection and data analyses were reported according to Reporting Recommendations for Tumour Marker Prognostic Studies (REMARK) criteria [18]. Research ethics approval was obtained from the respective local research ethics committees (KHP Cancer Biobank REC ref 18/EE/0025, Barts Cancer Institute REC ref 21/EE/0072 until January 2026, Medical Ethics Committee of Tianjin Medical University Cancer Institute and Hospital, Ek2020021) and the Institutional Review Board of the Netherlands Cancer Institute, clinical trial information: NCT03087409.

**Pathologist annotations**

A subset of 114 WSIs was selected from the overall dataset and split into training (*n* = 100), validation (*n* = 9), and test (*n* = 5) datasets to train and evaluate the supervised segmentation pipeline. To generate ground-truth pathologist labels at the pixel level, a single LN section on each WSI was manually annotated using QuPath version 0.3.0 [23].

**LN-detection algorithm**

To localise each individual LN section on a WSI, each slide was down sampled to a ×0.625 magnification. Artefacts were removed by filtering on the red range of the hue spectrum to highlight the H&E-stained LNs. To capture the LN coordinates, the filtered image was converted into grayscale, and four bilateral filters were applied to smooth the sharp edges of the surrounding adipose tissue by averaging pixel intensities, followed by two iterations of a hybrid Otsu thresholding algorithm [19]. Finally, a contouring algorithm was applied to the binary mask to localise each section on the slide.

**Tessellation and preprocessing of WSI**

For each WSI and ground-truth mask pair, a series of overlapping tiles are extracted, bounded by the coordinates of the LN contours (Figure 2B and supplementary material, Figure S1), at magnifications ×2.5, ×5, and ×10. For the MS U-Net model, the best performance was achieved training at an input of ×10 (effectively ×2.5, ×5, and ×10 combined) with a tile size of 1,024 × 1,024 and a sliding window of 512.

**Deep learning models**

For the segmentation of GCs and sinuses in LNs, we implemented three FCNs based on a U-Net architecture (referred to as U-Net) with symmetrical encoder-decoder paths. Details on model architectures can be found in supplementary material, Figure S1: (i) a standard U-Net consisting of an encoder that successively encodes a set of richer feature representations using five convolution blocks. The convolution blocks contain two convolution layers, each one followed by a batch normalisation layer. All convolution layers use a kernel size of 3×3 and are followed by a rectified linear unit (ReLu) activation layer. Between each convolution block, feature maps are downsampled spatially by a factor of two to continually increase the receptive field across the subsequent kernels. The number of filters, and therefore channels, is doubled at each successive convolution block from 32 to 512. Following a symmetrical design, the decoder contains five upsampling blocks with two convolutions and batch normalisation layers in each block. At the end of each block, the feature maps are upsampled using simple bilinear interpolation to reconstruct a segmented representation of the original image. Skip connections are added between encoder and decoder paths to propagate high-level feature information from the encoder to the decoder path, which aids the decoder in its localisation task; (ii) a U-Net model with an attention mechanism that upweights salient features during training (referred to as AttenU-Net) [21]. The AttenU-Net model extends the base U-Net by adding self-attention gates to each skip connection. The gates use features from the encoder path and a gating signal from higher-level activations in the encoder to learn attention coefficients during training. The attention weights give stronger influence on salient features that have strong activation responses in the intermediate layers during training and help to downweight features that are less important for performing the final segmentation; and (iii) a multiscale U-Net approach that assimilates semantic information from different scales during training using atrous convolutions [22] (referred to as MS U-Net). The MS U-Net model extends the base U-Net by replacing each convolution block with a multiscale module, shown in supplementary material, Figure S1, to encode information and semantic context from multiple resolutions into a single feature representation. The block first downsamples the resolution by a factor of two and a factor of four in parallel to create three activation maps of differing resolution. The three activation maps are passed through two convolution layers separately, where the first use dilated convolutions for the kernel to efficiently increase its receptive field. The output for the two lower-resolution maps is upsampled to the original dimensions and the features are combined into a single feature map combining information from across all three resolutions. In the decoder path, we pass the learned multiscale feature map through a decoder with the same architecture as the two previous models.

**Model training**

Models were trained from scratch and evaluated on pairs of tiles and ground-truth masks. The final MS U-Net model was trained on an input tile magnification of ×10 with a size of 1,024 × 1,024 (train, *n* = 11,324; validation, *n* = 2,694; test, *n* = 742). The number of training epochs was decided dynamically using an early stopping criterion based on the loss and dice curves of the validation set. A weighted cross-entropy loss was used to correct any bias towards the overrepresented class (background pixels); weights were calculated based on the inverse of the number of pixels within each class. Gradient updates were guided by an Adam optimiser with a constant learning rate of 0.0001. Data augmentation was applied to the tiles on the fly before training, including random flip, random rotation, and colour jitter (brightness, contrast, saturation, and hue). Pipelines were implemented with both PyTorch version 1.9.1 and Tensorflow version 2.7.0 deep learning libraries. Experiments were run on a high-performance computing (HPC) device using either the Nvidia GeForce RTX 2080 Ti or GTX 1080 Ti (NVIDIA Corporation, Santa Clara, CA, USA).

**Cancer cell detection**

To determine whether a LN from a known LN-positive breast cancer patient harboured metastasis, the Neural Conditional Random Field (NCRF) deep learning framework [20] was applied to 1,143 WSIs. A pathologist (ASh) manually checked all LNs with a positive cancer prediction result and confirmed their involved LN status.

**Morphological feature quantification**

For each WSI, the corresponding *smuLymphNet* prediction mask is split based on the set of contours generated in the LN-detection step. To characterise GCs, we captured (i) the number of GCs, (ii) the mean GC area, and (iii) the mean circularity of GCs. The average GC area, $A_{GC},$ per LN and the average circularity $A_{GC}$, is calculated using Equations 1 and 2:

$$\begin{aligned} \overline{A}_{\mathrm{GC}}=\frac{\sum_{i=1}^{N} {c^{2}A}_{,i}}{N_{\mathrm{GC}}} (1) \end{aligned}$$

$$\begin{aligned} \overline{C}_{\mathrm{GC}}=\frac{\sum_{i=1}^{N} \frac{\left( 4\pi A_{i} \right)}{{\mathrm{Perimeter}_{i}}^{2}}}{N_{\mathrm{GC}}}\left( 2 \right) \end{aligned}$$

Here, the area for a GC$, A_{i}$, is calculated using OpenCV version 4.1.2, $N_{\mathrm{GC}}$ is the total number of GCs, and *c* is the base resolution of the WSI, 0.2$3 \mu m/\mathrm{pixel}$, adjusted for the magnification level at which the analysis was performed. The GC perimeter was measured using the *arclength* function in OpenCV version 4.1.2 Python library. Given the continuous branching morphology of sinuses in LNs, we recorded the overall area of sinus tissue in a LN section given by Equation 3:

$$\begin{aligned} A_{\mathrm{sinus}}={c^{2}n}_{p}(3) \end{aligned}$$

Here, $n_{p}$ is the number of pixels classed as sinus within the prediction mask.

**Statistical analyses and outcome association**

Standard summary statistics were used to establish associations between morphometric immune features and patient outcomes. The primary endpoint was distant metastasis-free survival, defined as the date of first invasive recurrence or second primary tumour or death from any cause. We performed an iterative process to determine optimal cut-off points by a minimal *p* value approach (supplementary material, Figure S7) [25]. Kaplan–Meier methods were used to compare survival curves across groups. Cox regression proportional hazards models were performed to estimate the hazard ratios according to clinicopathological and histologically assessed features across all endpoints in univariate and multivariate analyses. The statistical significance of features was assessed using the log-likelihood test, whereby a two-sided *p* < 0.05 was considered significant. We used the statistical language R (version 4.1.1) to calculate the statistics [26].

**Interpathologist concordance**

A single LN on 24 WSIs was randomly selected for manual annotation by four pathologists (FL, ASh, SR, PG) (Figure 3A) using QuPath version 0.3.0 [23]. Ground-truth binary masks of these 24 pathologist-annotated LNs were compared for every pair of pathologists using the Dice coefficient.

**Subcapsular sinus quantification**

A heuristic method was implemented to calculate the width of the subcapsular sinus (SCS). Four points were selected based on a reference axis along the LN, chosen as the longest diameter across the LN section. Two points were determined as the intersection of the axis with the SCS on both sides and two at the intersection of the SCS based on a second axis orthogonal to the first. These four measurements were averaged to give the final indication of the SCS width (Equation 4) for each LN.

$$\begin{aligned} SCS width per node=\frac{w_{1}+w_{2}+w_{3}+w_{4}}{4}\left( 4 \right) \end{aligned}$$

where$w_{1,2,3,4}$ is the diameter at each of the selected points on the SCS.

**Supplementary Figures S1–S7**

**
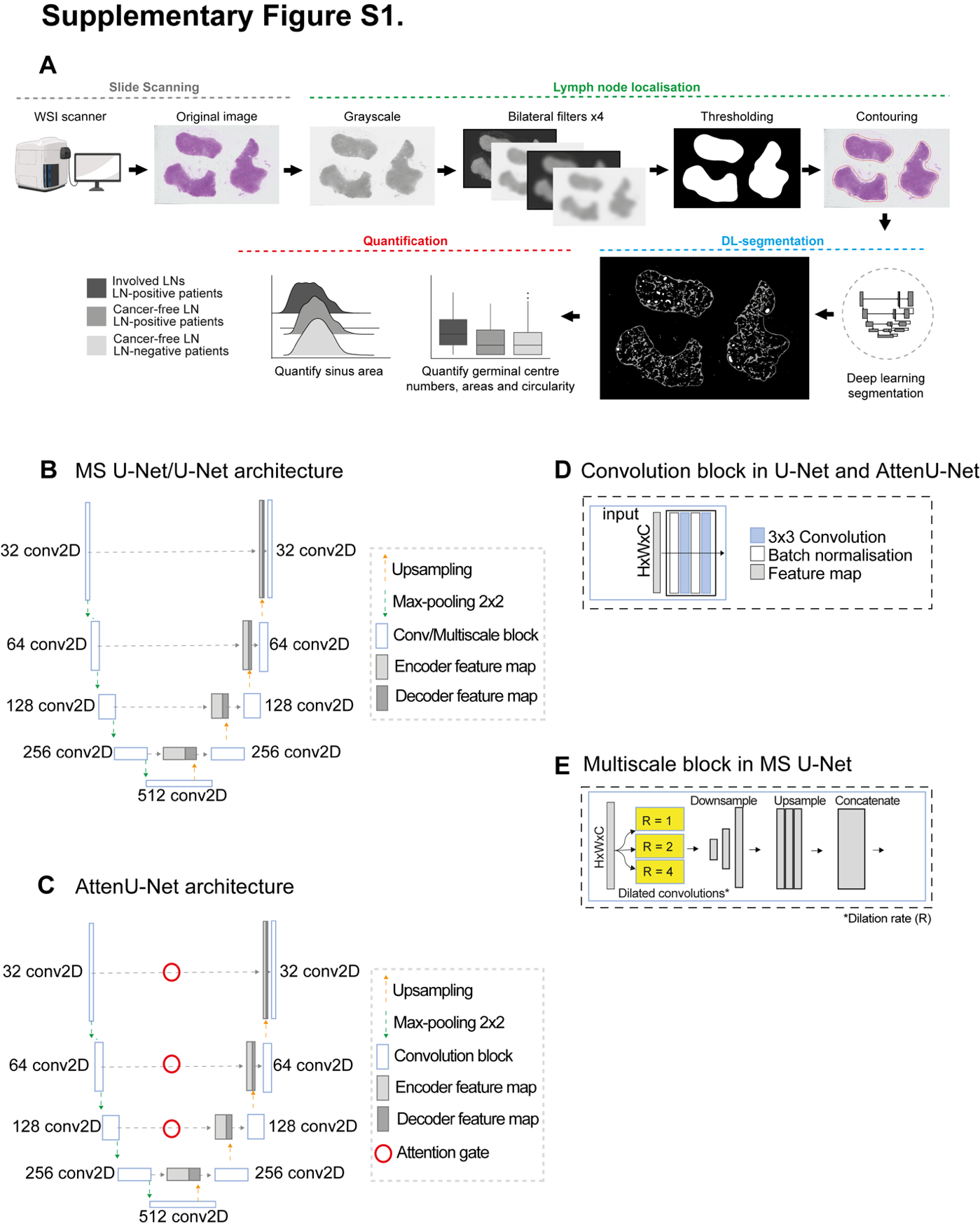
**

**Figure S1. *smuLymphNet* pipeline and network architecture for FCN models.** (A) Schematic representation of *smuLymphNe*t to detect each LN section from the background to segment GCs and sinuses and to perform quantification of these histological features. (B) Schematic representation of base model architecture for the three U-Net models. The models utilise an encoder-decoder structure, whereby the encoder has five convolution blocks. The decoder mirrors the encoder with a symmetrical design and is used to reconstruct the spatial information lost during feature extraction. (C) The second model architecture, AttenU-Net, extends this base architecture by adding self-attention gates to each skip connection to highlight salient image regions. (D) Convolution blocks were used in the first two models, which contain two convolution layers each with the same number of kernels and a size of 3×3. Each convolution layer is followed by a batch normalisation layer. (E) A multiscale module was used in the third model, MS U-Net. This extends the U-Net implementation by integrating a multiscale module into the encoder that assimilates semantic information from different scales whilst learning a feature representation of the input.


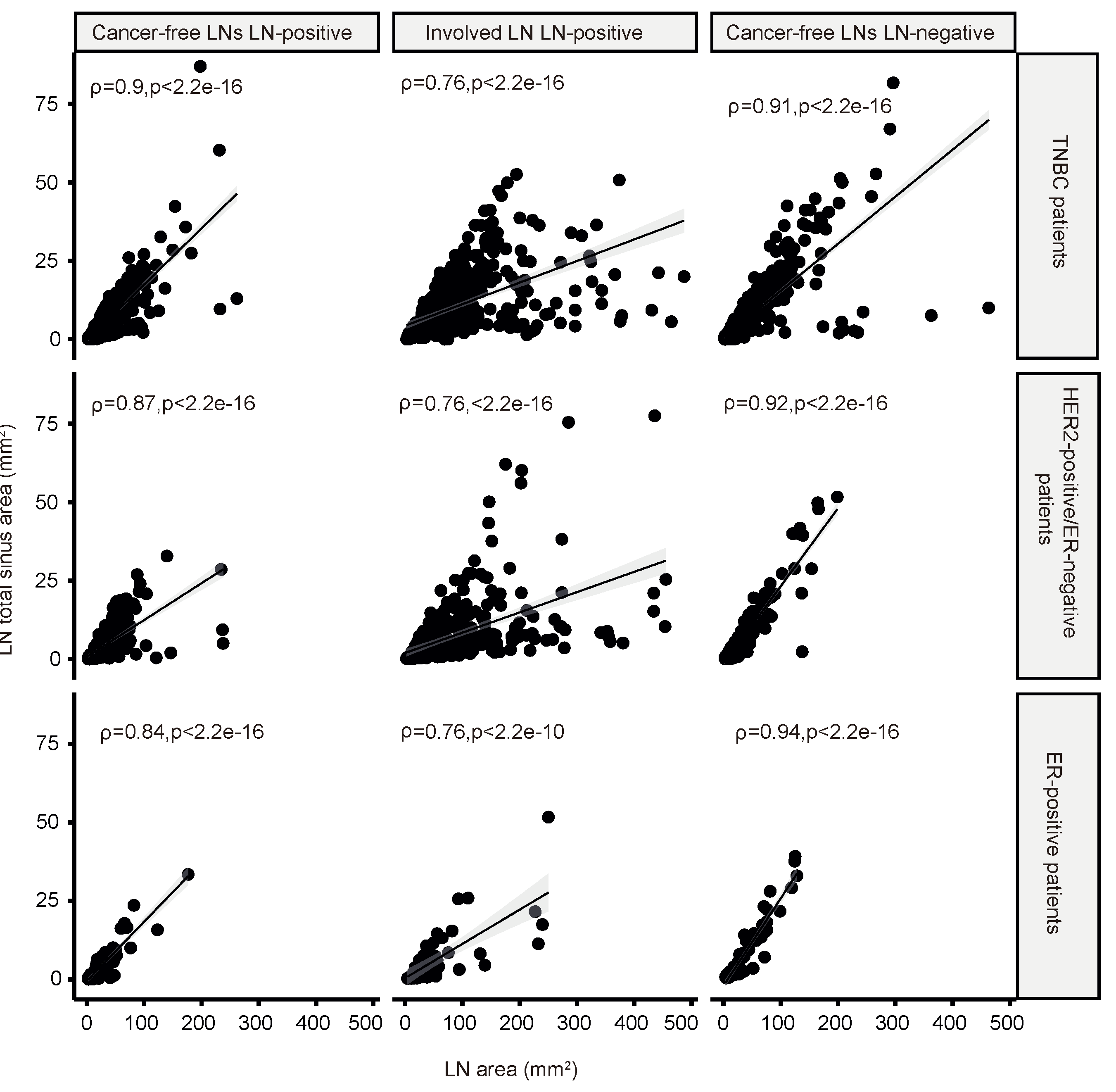


**Figure S2. Correlation between sinus area and total LN area.** Scatter plots show a positive correlation (rho > 0.7) between sinus areas and total LN areas across all histological subtypes: TNBC, HER2-positive/ER-negative, and ER-positive patients and LN status: involved LNs in LN-positive patients, cancer-free LNs in LN-positive patients, and cancer-free LNs in LN-negative patients.


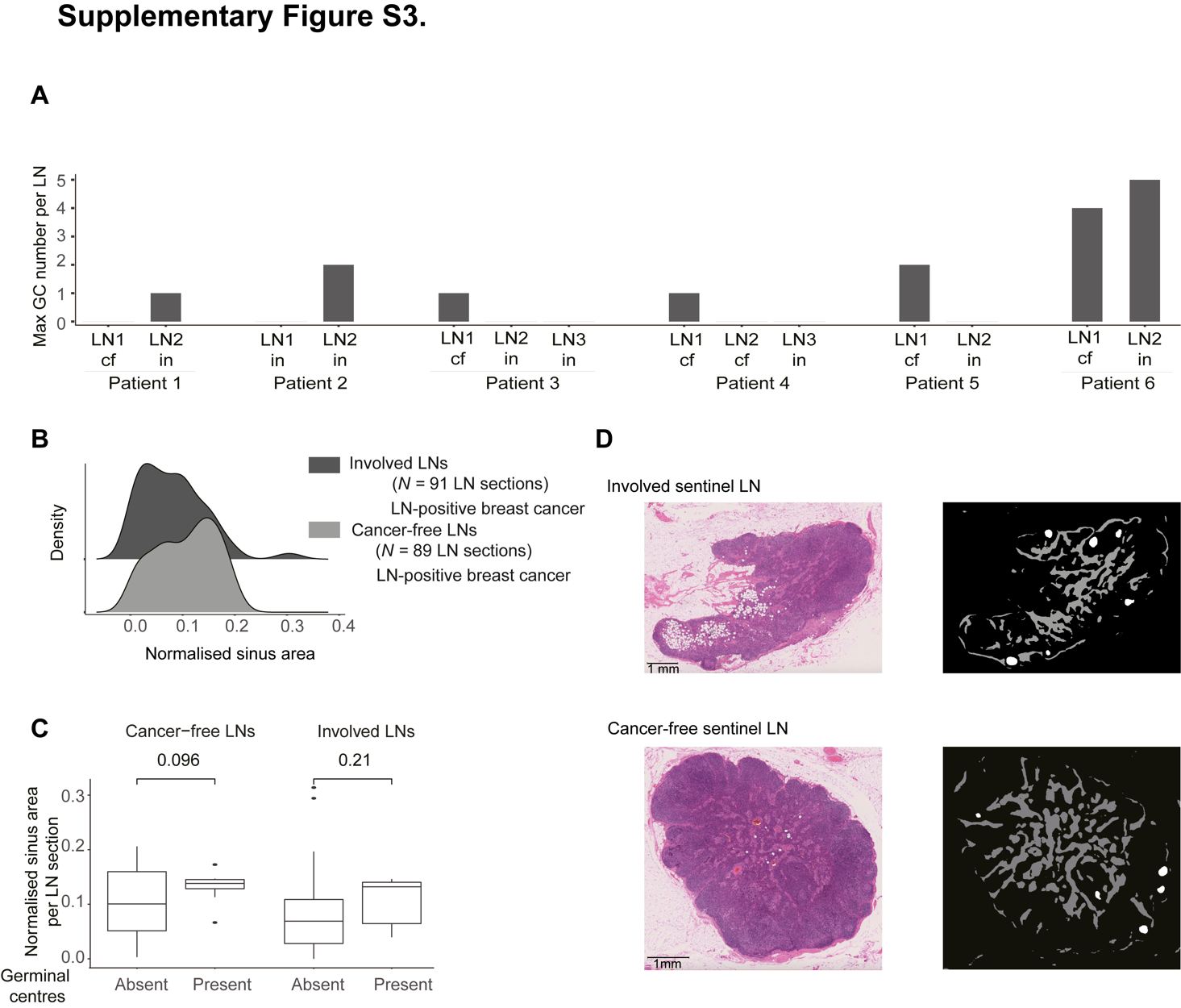


**Figure S3.** ***smuLymphNet*-captured GCs and sinuses, their quantification in sentinel LNs.** (A) The LN sections were separated into (i) involved LNs (in) and (ii) cancer-free LNs (cf) from LN-positive patients. Bar chart shows distribution of captured GCs within each LN in each patient; “in” means involved LN, “cf” means cancer-free LN. (B) Normalised sinus areas per LN were determined by capturing the total MS U-Net-based sinus area divided by the LN section area. Density plots show the distribution of normalised sinus area per LN section. Statistical significance was assessed by false discovery rate (FDR)-adjusted Kruskal–Wallis tests. (C) Boxplots showing normalised sinus area per LN section separated by LNs in which GCs were present or absent. Statistical significance was assessed by two-sided Wilcoxon rank sum test. (D) Example of MS U-Net model segmentation in involved and cancer-free sentinel LNs from a LN-positive breast cancer patient. A thumbnail of the H&E-stained sentinel LN section (left) and its MS U-Net model segmentation (right) are shown; sinus segmentations are in grey, GC segmentations in white.


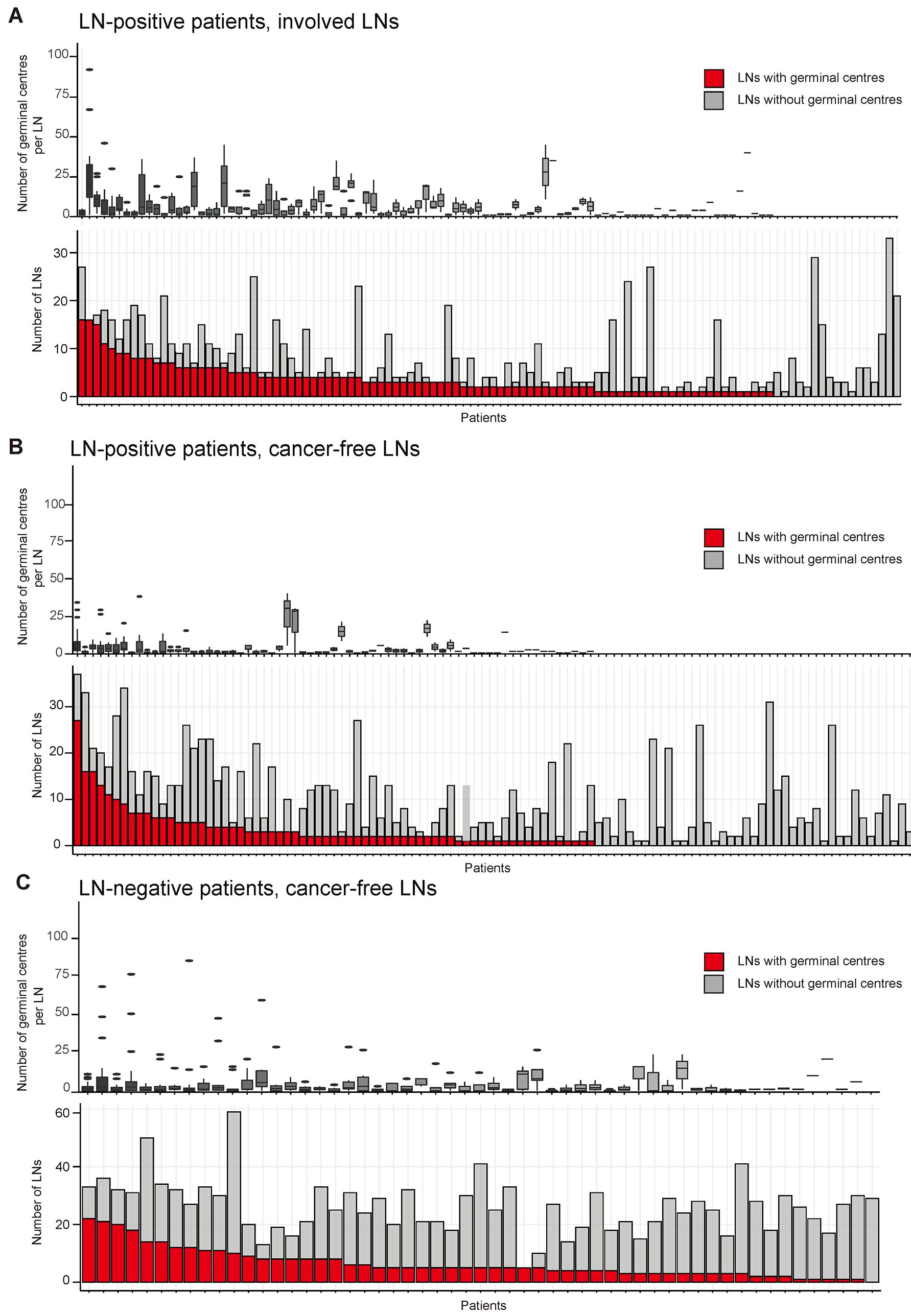


**Figure S4. Germinal centre counts per patient is independent of number of LNs assessed.** Top: boxplots show distribution of GCs per LN for each patient. Bottom: stacked boxplots show total number of LN sections split by number of LN sections with GCs (red) and without GCs (light grey) for each patient. Partitioned by (A) involved LNs from LN-positive TNBC patients, (B) cancer-free LNs from LN-positive TNBC patients, and (C) cancer-free LNs from LN-negative TNBC patients from Guy’s Hospital cohort. Points indicate outliers that are 1.5 times the interquartile range. Straight lines indicate LN with just a single germinal centre.


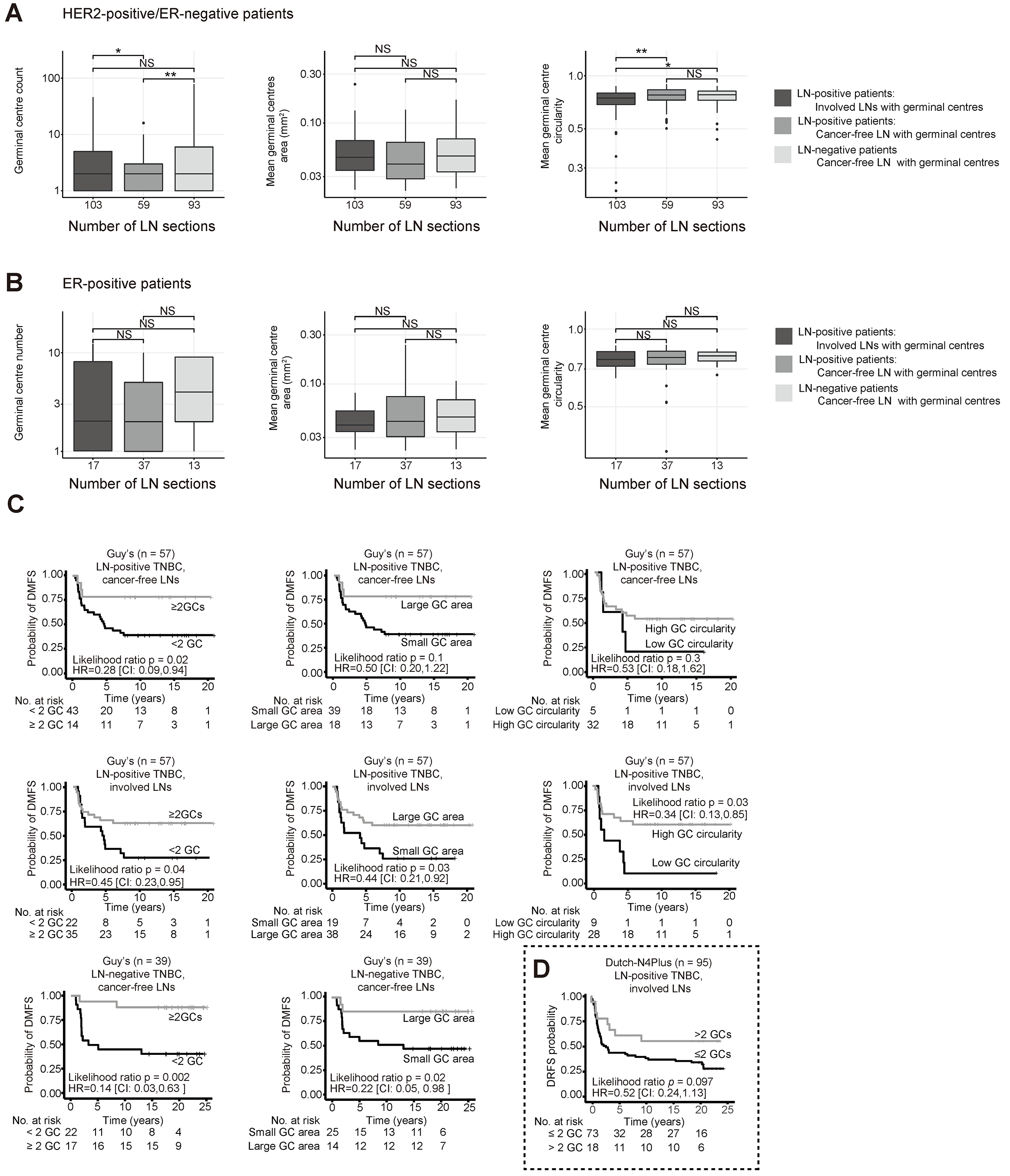


**Figure S5. GC quantitative assessment and outcome analyses.** Boxplots showing GC numbers, average GC area, average GC circularity per LN in (A) HER2-positive/ER-negative and (B) ER-positive breast cancer patients. LNs partitioned into involved LNs in LN-positive patients, cancer-free LNs in LN-positive patients, and cancer-free LNs in LN-negative patients. Wilcoxon rank sum test (* *p* ≤ 0.05, ** *p* ≤ 0.01, NS = not significant), (C) Kaplan–Meier curves showing distant metastasis-free survival (DMFS) for TNBC patients from Guy’s Hospital. Patients were dichotomised into those with on average <2 GCs or ≥2 GCs per LN, GC average area >0.015 mm^2^ or ≤0.015 mm^2^ per LN, GC average circularity >0.67 or ≤0.67 per LN (patients with no GCs in the assessed LNs were removed when analysing circularity and average area) across all assessed involved or cancer-free LNs of LN-positive (*n* = 57) and LN-negative TNBC (*n* = 39) patients of Guy’s Hospital. Of note, Kaplan–Meier analysis could not be performed in cancer-free LNs in LN-negative TNBC since all GCs were assigned to the high circularity group. (D) Kaplan–Meier curve of distant recurrence-free survival (DRFS) for TNBC patients of the Dutch-N4plus trial (*n*=95). Patients were dichotomised into those with >2 GCs or $\leq$2 GCs across all assessed involved LNs. *P* values correspond to likelihood ratio tests. Hazard ratio (HR), 95% confidence interval (95% CI) are listed.

**
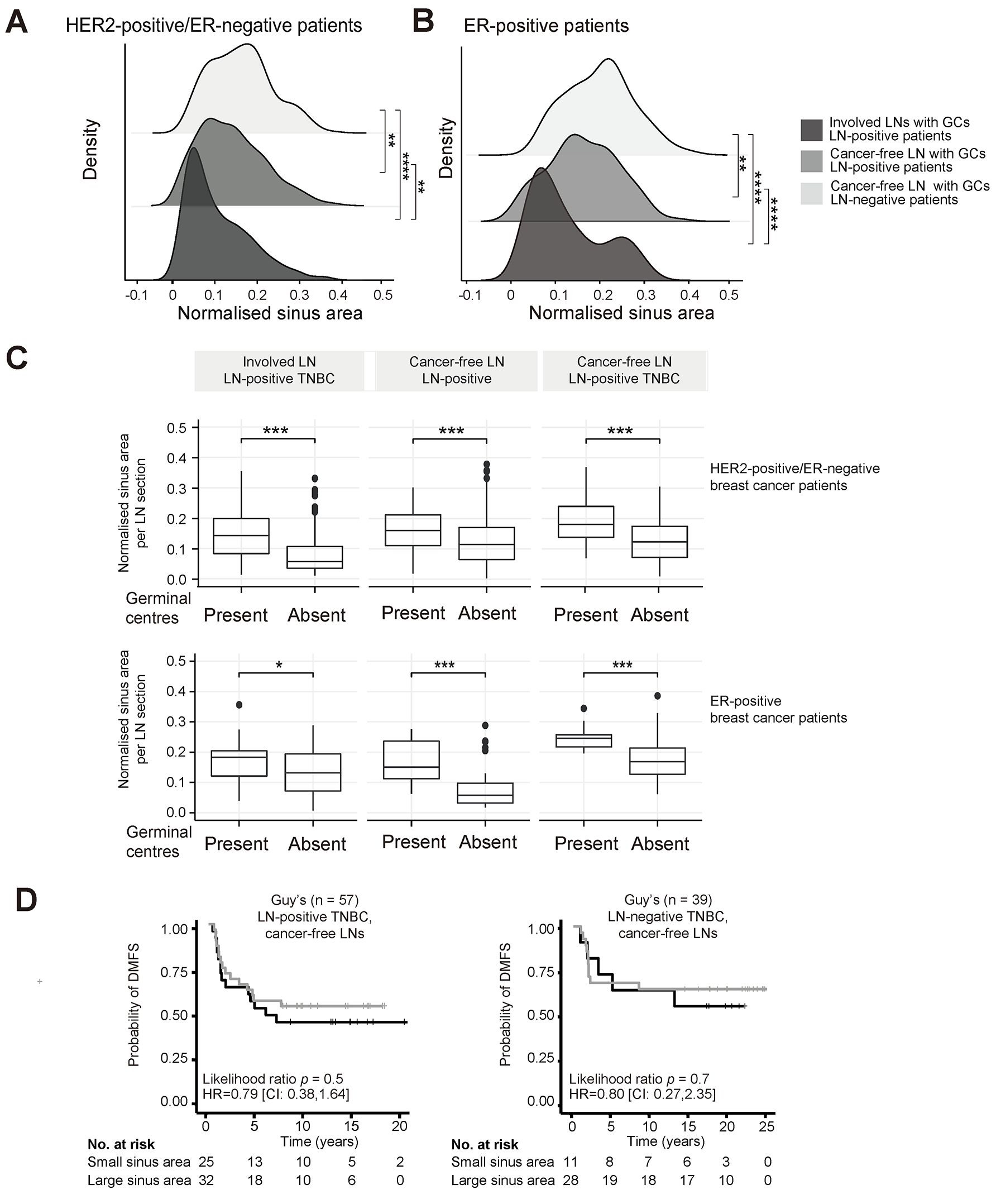
**

**Figure S6. Sinus area, quantitative assessment, and outcome analyses.** Density plot of normalised sinus area per LN section in (A) HER2-positive/ER-negative and (B) ER-positive breast cancer patients. Partitioned into cancer-free LNs from LN-negative patients, involved LNs from LN-positive patients, cancer-free LNs from LN-positive patients. Boxplots showing normalised sinus area per LN section in HER2-positive/ER-negative and ER-positive in cancer-free LNs from LN-negative patients, involved LNs from LN-positive patients, cancer-free LNs from LN-positive patients. (C) Boxplots showing normalised sinus area per LN section in HER2-positive/ER-negative and ER-positive in cancer-free LNs from LN-negative patients, involved LNs from LN-positive patients, cancer-free LNs from LN-positive patients, dichotomised based on whether GCs were present or absent. Wilcoxon rank sum test (** *p* ≤ 0.01, ****p* ≤ 0.001, *****p* ≤ 0.0001). (D) Kaplan–Meier analyses of distant metastasis-free survival (DMFS) for TNBC patients of Guy’s Hospital cohort. Patients were dichotomised into those with normalised sinus area >0.13 mm^3^(large sinus area) or ≤0.13 mm^2^ (small sinus area) in cancer-free LNs of LN-positive (*n* = 57) and LN-negative TNBC (*n* = 39) patients of Guy’s Hospital.


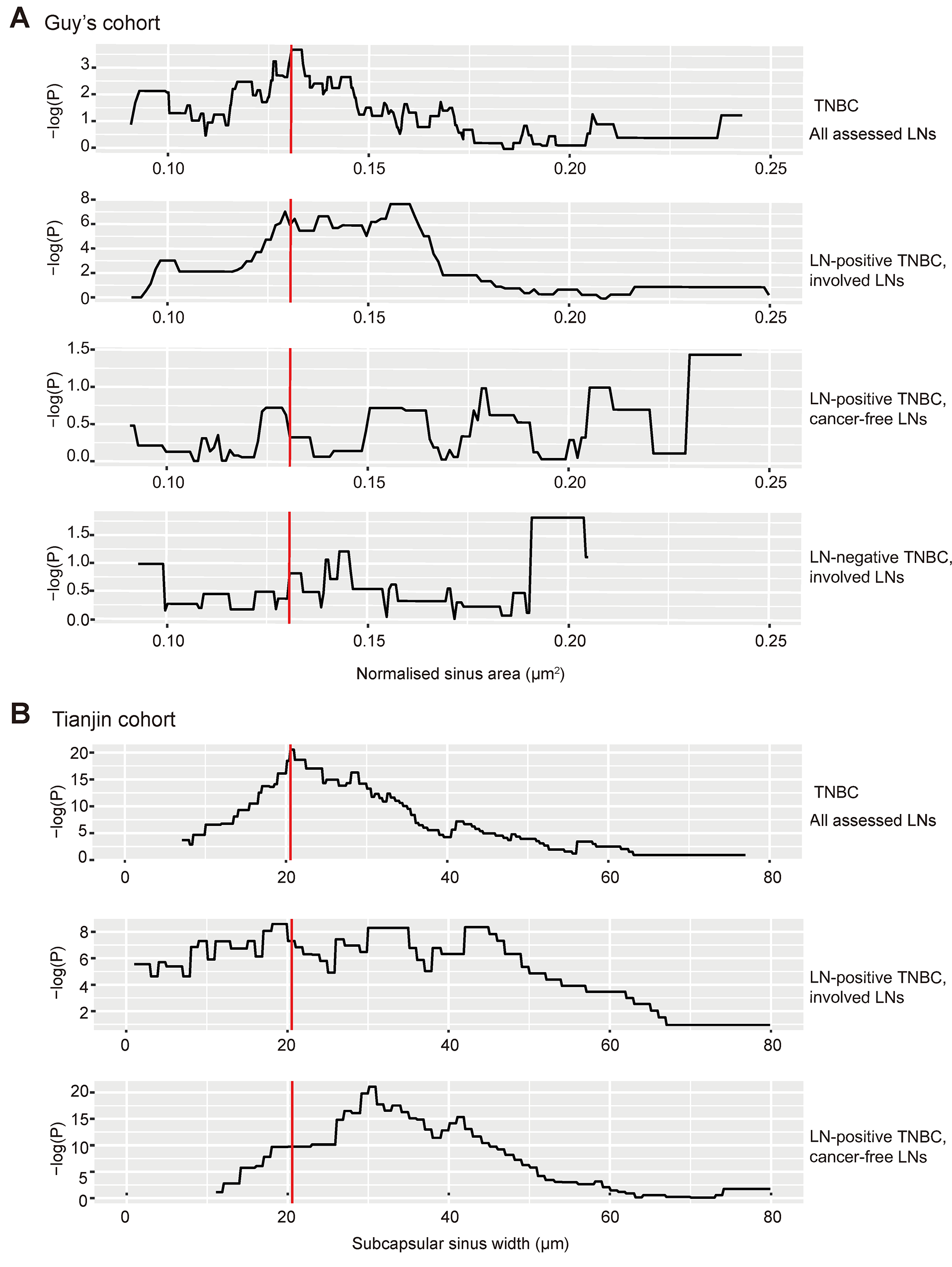


**Figure S7. Defining prognostic sinus area cut-off**. The optimal normalised sinus area cut-off was determined by taking all patients and applying an iterative process, using a minimal *p* value approach. For the Guy’s Hospital cohort, the line charts show a series of normalised sinus area cut-off values and the *p* value when that cut-off value was applied in (i) all assessed LNs in TNBC patients, (ii) involved LNs in LN-positive TNBC, (iii) cancer-free LNs in LN-positive TNBC, and (iv) cancer-free LNs in LN-negative TNBC. An optimal cut-off was at 0.13 in Guy’s cohort. For the Tianjin cohort, an optimal cut-off was at 20 mm for SCS width.

Table S1. Clinical characteristics of patients from Dutch-N4Plus TNBC cohort (*N*=95).

All patients sTILs

sTILs

Conventional

High-dose

***BRCA1*-like scars**

Age (years)

< **20%**

≥ **20%**

treatment

treatment No Yes

| Mean (SD) | 42.3 (6.66) | 41.4 (5.63) | 43.1 (6.76) | 42.0 (7.10) | 42.7 (6.28) | 44.1 (6.46) | 40.9 (6.54) |
| --- | --- | --- | --- | --- | --- | --- | --- |
| Median [Min,Max] | 43.0 [26,53] | 41.0 [33,53] | 44.0 [28,53] | 43 [26,53] | 42 [31,53] | 45 [26,53] | 41 [28,52] |

Tumour size

| T1 | 25 (26.3%) | 5 (17.2%) | 17 (30.9%) | 12 (26.1%) | 13 (26.5%) | 13 (30.2%) | 12 (23.1%) |
| --- | --- | --- | --- | --- | --- | --- | --- |
| T2 | 55 (57.9%) | 14 (48.3%) | 34 (61.8%) | 27 (58.7%) | 28 (57.1%) | 22 (51.2%) | 33 (63.5%) |
| T3 | 14 (14.7%) | 10 (34.5%) | 4 (7.3%) | 7 (15.2%) | 7 (14.3%) | 8 (18.6%) | 6 (11.5%) |
| NA | 1 (1.1%) | 0 (0%) | 0 (0%) | 0 (0%) | 1 (2.0%) | 0 (0%) | 1 (1.9%) |
| **Number of involved** | | | | | | | |
| **lymph nodes** |  |  |  |  |  |  |  |
| <10 N+ | 58 (61.1%) | 17 (58.6%) | 34 (61.8%) | 27 (58.7%) | 31 (63.3%) | 24 (55.8%) | 34 (65.4%) |
| ≥10 N+ | 37 (38.9%) | 12 (41.4%) | 21 (38.2%) | 19 (41.3%) | 18 (36.7%) | 19 (44.2%) | 18 (34.6%) |
| **Bloom–Richardson grade** | | | | | | | |
| Grade I | 3 (3.2%) | 1 (3.4%) | 1 (1.8%) | 1 (2.2%) | 2 (4.1%) | 2 (4.7%) | 1 (1.9%) |
| Grade II | 18 (18.9%) | 8 (27.6%) | 7 (12.7%) | 11 (23.9%) | 7 (14.3%) | 11 (25.6%) | 7 (13.5%) |
| Grade III | 71 (74.7%) | 20 (69.0%) | 44 (80%) | 33 (71.7%) | 38 (77.6%) | 28 (65.1%) | 43 (82.7%) |
| NA | 3 (3.2%) | 0 (0%) | 3 (5.5%) | 1 (2.2%) | 2 (4.1%) | 2 (4.7%) | 1 (1.9%) |
| **sTILs** |  |  |  |  |  |  |  |
| <20% | 29 (30.5%) | 29 (100%) | 0 (0%) | 15 (32.6%) | 14 (28.6%) | 15 (34.9%) | 14 (26.9%) |
| ≥20% | 55 (57.9%) | 0 (0%) | 55 (100%) | 27 (58.7%) | 28 (57.1%) | 21 (48.8%) | 34 (65.4%) |
| NA | 11 (11.6%) | 0 (0%) | 0 (0%) | 4 (8.7%) | 7 (14.3%) | 7 (16.3%) | 4(7.7%) |
| **Treatment protocol** |  |  |  |  |  |  |  |
| Conventional | 46 (48.4%) | 15 (51.7%) | 27 (49.1%) | 46 (100%) | 0 (0%) | 16 (37.2%) | 30 (57.7%) |
| High-dose | 49 (51.6%) | 14 (48.3%) | 28 (50.9%) | 0 (0%) | 49 (100%) | 27 (62.8%) | 22 (42.3%) |
| ***BRCA1*-like scar** |  |  |  |  |  |  |  |
| No | 43 (45.3%) | 15 (51.7%) | 21 (38.2%) | 16 (34.8%) | 27 (55.1%) | 43 (100%) | 0 (0%) |
| Yes | 52 (54.7%) | 14 (48.3%) | 34 (61.8%) | 30 (65.2%) | 22 (44.9%) | 0 (0%) | 52 (100%) |
| **DRFS** |  |  |  |  |  |  |  |
| No event | 33 (34.7%) | 5 (17.2%) | 25 (45.4%) | 14 (30.4%) | 19 (38.8%) | 14 (32.6%) | 19 (36.5%) |
| Event | 62 (65.3%) | 24 (82.8%) | 30 (54.5%) | 32 (69.6%) | 30 (61.2%) | 29 (67.4%) | 33 (63.5%) |
| **DRFS time days** |  |  |  |  |  |  |  |
| Mean (SD) | 1420 (1980) | 999 (1580) | 1660 (2100) | 1570 (2250) | 1270 (1670) | 1780 (2460) | 1110 (1400) |
| Median [Min,Max] 556 [15,7330] 473 [25,7230] 647 [15,7330] 579 [15,7330] 516 [25,7320] 576 [15,7330] 536 [25,5520] | | | | | | | |
| NA | 33 (34.7%) | 5 (17.2%) | 25 (45.5%) | 14 (30.4%) | 19 (38.8%) | 14 (32.6%) | 19 (36.5%) |

Table S2. Clinical characteristics and immune features in LNs of patients from Tianjin TNBC cohort.

**A. Clinical characteristics**

|  | | Tianjin TNBC cohort, *N* = 99 (%) | Tianjin TNBC cohort  (sinus assessment available),  *N* = 85 (%) | | P value | |
| --- | --- | --- | --- | --- | --- | --- |
| sTILs  <20%  ≥20%  Tertiary lymphoid structures | | 52 (52) | 47 (55) | |  | |
|  |  | 47 (48) | 38 (45) | | 0.707^a^ | |
| Absent | | 77 (78) | 65 (76) | |  | |
| Present | | 22 (22) | 20 (24) | | 0.833^a^ | |
| Age at diagnosis | |  |  | |  | |
| <50 | | 46 (46) | 41 (48) | |  | |
| ≥50 | | 53 (54) | 44 (52) | | 0.810^a^ | |
| Tumour size | |  |  | |  | |
| pT1 | | 22 (22) | 17 (20) | |  | |
| pT2 | | 70 (71) | 62 (73) | |  | |
| pT3 | | 6 (6) | 5 (6) | |  | |
| pT4 | | 1 (1) | 1 (1) | | 0.985^a^ | |
| Histological grade | |  |  | |  | |
| III | | 99 (100) | 85 (100) | |  | |
| Lymphovascular invasion | |  |  | |  | |
| Absent | | 38 (38) | 31 (36) | |  | |
| Present | | 61 (62) | 54 (64) | | 0.789^a^ | |
| Lymph node status | |  |  | |  | |
| pN1 (1–3) | | 63 (64) | 53 (63) | |  | |
| pN2 (4–9) | | 21 (21) | 18 (21) | |  | |
| pN3 (>10) | | 15 (15) | 14 (16) | | 0.969^a^ | |
| Distant metastasis  Absent  Present  Breast cancer-specific death | | 73 (74) | 59 (69) | |  | |
|  |  | 26 (26) | 26 (31) | | 0.516^a^ | |
| Absent | | 80 (81) | 66 (78) | |  | |
| Present | 19 (20) | | 19 (22) | | 0.597^a^ | |

^a^ Chi-squared test

**B. Immune features in LNs**

|  | Tianjin TNBC cohort, *N* = 99 (%) | Tianjin TNBC cohort  (sinus assessment available),  *N* = 85 (%) | P value |
| --- | --- | --- | --- |
| LN assessment |  |  |  |
| Cancer-free LNs, median (range) | 16 (3–31) | 16 (3–31) |  |
| Involved LNs, median (range) | 2 (1–18) | 2 (1–8) |  |
| GC assessment in LNs per patient |  |  |  |
| All LNs, *N* (%) |  |  |  |
| GC absent | 6 (6.1) | 6 (7.1) |  |
| GC present | 93 (93.9) | 79 (92.9) | 0.785^a^ |
| Cancer-free LNs | 7 |  |  |
| GC NA | 1 | 0 |  |
| GC absent | 13 (13.3) | 13 (15.3) |  |
| GC present | 85 (86.7) | 72 (88.2) | 0.695^a^ |
| Involved LNs |  |  |  |
| GC NA | 9 (9.1) | 0 |  |
| GC absent | 18 (18.2) | 17 (20) |  |
| GC present | 72 (72.7) | 68 (80) | 1a |
| Total number of GCs  across all assessed LNs per patient  Cancer-free LN, median (range)  Involved LN, median (range)  Max GC number in a LN  across all assessed LNs per patient | 9 (1–175) | 8 (0–175) |  |
|  | 7 (0–214) | 7 (0–214) | 0.870^a^ |
| Cancer-free LN, median (range) | 5 (0–63) | 5 (0–63) |  |
| Involved LN, median (range) | 6 (0–54) | 6 (0–54) | 1a |
| Average GC number |  |  |  |
| Cancer-free LN, median (range) | 3 (0–35) | 3 (0–17) |  |
| Involved LN, median (range) | 5 (0–43) | 5 (0–43) | 1a |
| Average subcapsular sinus (SCS) width |  |  |  |
| Cancer-free LN, median (range) | 47 (11–108) | 44 (11–108) |  |
| Involved LN, median (range) | 22 (0–82) | 22 (0–82) | 0.857^a^ |

^a^ Chi-squared test

**Table S3.** Univariate and multivariate Cox proportional hazard analyses in Dutch-N4Plus TNBC cohort.

**A. Analyses for GCs across all assessed lymph nodes (LNs).**

| **Dutch-N4Plus TNBC cohort** |  | **Model P** | **Hazard ratio** | **95% CI** |
| --- | --- | --- | --- | --- |
| **Univariate Distant Recurrence-Free Survival** |  |  |  |  |
| LN-positive TNBC (*N* = 95), involved LNs  Total GC number per patient (≤2 versus >2) |  | 0.097 | 0.52 | 0.24–1.13 |
| pTStage |  | 0.21 | 1.31 | 0.86–2.01 |
| sTILs (<20% versus ≥20%) |  | 0.004 | 0.42 | 0.23–0.75 |
| Bloom–Richardson grade |  | 0.52 | 1.99 | 0.24–16.2 |
| Number of involved LNs (<10 versus ≥10) |  | 0.1 | 0.65 | 0.39–1.08 |
|  | **Covariate P** | **Model P** | **Hazard ratio** | **95% CI** |
| **Multivariate Distant Recurrence-Free Survival** |  |  |  |  |
| Adjusted for sTILs (<20% versus ≥20%) |  |  |  |  |
| LN-positive TNBC (*N* = 95), involved LNs |  |  |  |  |
| Total GC number (≤2 versus >2) | 0.36 | 0.145 | 0.38 | 0.05–3.00 |

Statistical significance was assessed using likelihood ratio tests.

**B. Analyses for maximal normalised sinus area across all assessed lymph nodes (LNs).**

| **Dutch-N4Plus TNBC cohort** |  | **Model P** | **Hazard ratio** | **95% CI** |
| --- | --- | --- | --- | --- |
| **Univariate Distant Recurrence-Free Survival** |  |  |  |  |
| LN-positive TNBC (*N* = 95), involved LNs  Max normalised sinus area (≤0.13 mm^2^ versus >0.13 mm^2^) |  | 0.024 | 0.44 | 0.22–0.90 |
| pTStage |  | 0.21 | 1.31 | 0.86–2.01 |
| sTILs (<20% versus ≥20%) |  | 0.004 | 0.42 | 0.23–0.75 |
| Bloom Richardson grade |  | 0.52 | 1.99 | 0.24–16.2 |
| Number of involved LNs (<10 versus ≥10) |  | 0.1 | 0.65 | 0.39–1.08 |
|  | **Covariate P** | **Model P** | **Hazard ratio** | **95% CI** |
| **Multivariate Distant Recurrence-Free Survival** |  |  |  |  |
| Adjusted for sTILs (< 20% versus ≥ 20%) |  |  |  |  |
| LN-positive TNBC (*N* = 95), involved LNs |  |  |  |  |
| Max normalised sinus area (≤ 0.13 mm^2^ versus > 0.13 mm^2^) | 0.056 | 0.043 | 0.50 | 0.25–1.02 |
|  |  |  |  |  |

Statistical significance was assessed using likelihood ratio tests.
